# Phenotypic and genomic assessment of the potential threat of human spaceflight-relevant *Staphylococcus capitis* isolates under stress conditions

**DOI:** 10.3389/fmicb.2022.1007143

**Published:** 2022-11-03

**Authors:** Katharina Siems, Katharina Runzheimer, Anna Rehm, Oliver Schwengers, David Heidler von Heilborn, Liv Kaser, Franca Arndt, Claudio Neidhöfer, Jan Philipp Mengel, Marijo Parcina, André Lipski, Torsten Hain, Ralf Moeller

**Affiliations:** ^1^Department of Radiation Biology, Institute of Aerospace Medicine, German Aerospace Center (DLR), Cologne, Germany; ^2^Department of Bioinformatics and Systems Biology, Justus Liebig University Giessen, Giessen, Germany; ^3^Institute of Nutritional and Food Sciences, Food Microbiology and Hygiene, University of Bonn, Bonn, Germany; ^4^Institute for Medical Microbiology, Immunology and Hygiene, University Hospital of Cologne, Cologne, Germany; ^5^Institute of Medical Microbiology, Immunology and Parasitology, University Hospital Bonn, Bonn, Germany; ^6^Institute of Medical Microbiology, Justus Liebig University Giessen, Giessen, Germany; ^7^German Center for Infection Research (DZIF), Justus Liebig University Giessen, Partner Site Giessen-Marburg-Langen, Giessen, Germany

**Keywords:** space microbiology, coagulase-negative staphylococci, *Staphylococcus capitis*, bacterial stress response, radiation

## Abstract

Previous studies have reported that spaceflight specific conditions such as microgravity lead to changes in bacterial physiology and resistance behavior including increased expression of virulence factors, enhanced biofilm formation and decreased susceptibility to antibiotics. To assess if spaceflight induced physiological changes can manifest in human-associated bacteria, we compared three spaceflight relevant *Staphylococcus capitis* isolates (DSM 111179, ISS; DSM 31028, clean room; DSM 113836; artificial gravity bedrest study) with the type strain (DSM 20326^T^). We tested the three strains regarding growth, colony morphology, metabolism, fatty acid and polar lipid pattern, biofilm formation, susceptibility to antibiotics and survival in different stress conditions such as treatment with hydrogen peroxide, exposure to desiccation, and irradiation with X-rays and UV-C. Moreover, we sequenced, assembled, and analyzed the genomes of all four strains. Potential genetic determinants for phenotypic differences were investigated by comparative genomics. We found that all four strains show similar metabolic patterns and the same susceptibility to antibiotics. All four strains were considered resistant to fosfomycin. Physiological differences were mainly observed compared to the type strain and minor differences among the other three strains. The ISS isolate and the bedrest study isolate exhibit a strong delayed yellow pigmentation, which is absent in the other two strains. Pigments were extracted and analyzed by UV/Vis spectroscopy showing characteristic carotenoid spectra. The ISS isolate showed the highest growth rate as well as weighted average melting temperature (WAMT) of fatty acids (41.8°C) of all strains. The clean room isolate showed strongest biofilm formation and a high tolerance to desiccation. In general, all strains survived desiccation better in absence of oxygen. There were no differences among the strains regarding radiation tolerance. Phenotypic and genomic differences among the strains observed in this study are not inevitably indicating an increased virulence of the spaceflight isolate. However, the increased growth rate, higher WAMT and colony pigmentation of the spaceflight isolate are relevant phenotypes that require further research within the human spaceflight context. We conclude that combining genetic analysis with classical microbiological methods allows the detailed assessment of the potential threat of bacteria in highly regulated and extreme environments such as spaceflight environments.

## Introduction

Within prolonged space missions, astronauts are exposed to extreme conditions including microgravity, higher exposure to radiation, disturbed sleeping rhythms, stress, insufficient nutrition, and microbial contamination (Mermel, 2013). Exposure to these conditions may compromise the health of astronauts, considering the reduced immune function during spaceflight (Sonnenfeld and Shearer, 2002). However, space is not only an extreme environment for astronauts but also for microorganisms. The environmental conditions during spaceflight lead to changes of the astronaut’s skin and gut microbiome, as well as changes to the microbial composition of the built environment such as the International Space Station (ISS; Ilyin, 2005; Garrett-Bakelman et al., 2019; Voorhies et al., 2019), which is highly influenced by the astronaut’s microbiome itself (Avila-Herrera et al., 2020). In addition to the external influences of space (e.g., microgravity, radiation), there are also internal factors inside the station, which can be challenging for microorganisms. One important factor is the exposure to desiccation and nutrient depletion once microorganisms are deposited on surfaces through the air or through direct skin contact. Additionally, the surfaces inside the ISS are frequently cleaned and disinfected by using hydrogen peroxide containing wipes (Viroxtechnologies, 2018), which additionally puts stress upon microorganisms that are present on surfaces. Constant exposure to these stress conditions can alter microbial physiology and lead to the development of resistance mechanisms. One resistance mechanism in microorganisms is the production of pigments. However, to which extent increased pigmentation aids microbial survival within the spaceflight environment remains unclear and needs further research.

In general, the microbiome of spaceflight-relevant settings such as the ISS appears to be dominated by human-associated microorganisms (Venkateswaran et al., 2014; Mora et al., 2019). It was already shown that certain skin bacteria (e.g., *Streptococcus*, *Staphylococcus*, and *Corynebacterium*) become more abundant inflight (Voorhies et al., 2019). Regarding physiological adaptations that could threaten human health, some studies report no relevant changes in physiology (Mora et al., 2019) or even a decrease in virulence (Hammond et al., 2013). Nevertheless, other studies show increased expression of virulence factors and enhanced biofilm formation in simulated microgravity (Nickerson et al., 2000; Mauclaire and Egli, 2010) as well as increased surface interaction in microbial spaceflight isolates (Mora et al., 2019). Furthermore, some bacteria were reported to be less susceptible to antibiotics in microgravity or simulated microgravity (Taylor and Sommer, 2005; Tirumalai et al., 2019).

A bacterial species that is frequently isolated from spaceflight-relevant settings, for example inside the ISS, and also in clean room facilities, is *Staphylococcus capitis* (*S. capitis*; La Duc et al., 2007; Probst et al., 2010; Moissl-Eichinger et al., 2015; Sobisch et al., 2019). This species belongs to the coagulase-negative staphylococci (CoNS) that are considered beneficial skin colonizers, since they generate an acidic pH environment that can inhibit the growth of pathogenic microorganisms (Grice and Segre, 2011). Nevertheless, many CoNS are also considered opportunistic pathogens and have been frequently found in bloodstream infections in intensive care units (Cameron et al., 2015), also causing nosocomial late-onset sepsis in premature newborns (Wirth et al., 2020). The most important virulence factors of CoNS are their ability to form resistant biofilms on medical devices such as catheters and the high resistance of some strains to several antibiotics (Cui et al., 2013; Wirth et al., 2020; Manandhar et al., 2021). What separates *S. capitis* strains from other staphylococcal strains is primarily their colony morphology, cell wall composition, and their distinct carbohydrate metabolism (Kloos and Schleifer, 1975). *Staphylococcus capitis* can be subdivided into two subspecies: *S. capitis* subsp. *capitis* and *S. capitis* subsp. *urealyticus.* The main differences between the two are the positive urease activity, aerobic acid production from maltose, their fatty acid profile, and the different colony morphology (Bannerman and Kloos, 1991). In this study, we chose *S. capitis* subsp. *capitis* as model organism to determine the effect of spaceflight conditions on a human-associated bacterial species. One reason for that is that *S. capitis* has been detected as part of the ISS indoor microbiome, which is mainly known through metagenomic studies but also through cultivation approaches (Schiwon et al., 2013; Mora et al., 2019; Sobisch et al., 2019). Even though it is vital to know the composition of the microbiome of the ISS, little is known about the exact changes in this species’ physiology in the challenging conditions they face within the spaceflight environment. Furthermore, we found that *S. capitis* subsp. *capitis* is a suitable model organism for this type of study as it is known to be a biofilm former, is classified as a biosafety level 1 organism and is closely related to other clinically relevant CoNS.

In this study, three spaceflight-relevant isolates of *S. capitis* subsp. *capitis* were compared to the type strain. These include, one isolate from the ISS (Sobisch et al., 2019; DSM111179), one isolate from a clean room facility (DSM 31028), and one isolate from an artificial gravity bedrest study (DSM 113836). The type strain of the German Collection of Microorganisms and Cell Cultures (DSMZ; DSM 20326^T^) was isolated from human skin in 1975 (Kloos and Schleifer, 1975). The goal of this study is to unravel changes within the four isolates that might indicate adaptations toward space relevant conditions. For this, the four strains were compared regarding their phenotype by investigating growth and colony morphology on different media, metabolism, biofilm formation, susceptibility for antibiotics, and pigmentation. The genomes of the four strains were sequenced and used for comparative genomic analysis. Additionally, the strains were tested for their response to stress conditions by treatment with hydrogen peroxide (H_2_O_2_), irradiation with X-rays and UV radiation at 254 nm (UV-C) as well as desiccation under different atmospheric conditions.

## Materials and methods

### *Staphylococcus capitis* strains

In this work, we included four different *S. capitis* strains from spaceflight relevant settings (Table 1). Two of the strains were obtained from the DSMZ (D2^T^, D3). Strain K1 was isolated from V2A steel in a material exposure experiment aboard the ISS (Sobisch et al., 2019). H17 was isolated from a forehead skin swab that was taken from one of 12 test subjects in the bedrest study AGBRESA (Artificial Gravity Bed Rest—European Space Agency) at the Institute for Aerospace medicine at the German Aerospace Center (DLR, Cologne, Germany). In this study, the subjects experienced a 6° head down tilt for 60 days in order to simulate fluidic shifts similar to the shift observed in astronauts during spaceflight. Additionally, a human-centrifuge was tested as possible counter-measure against these fluidic shifts (Frett et al., 2020). The strain H17 was isolated at day 57 of the head down tilt phase.

**Table 1:** List of strains that were used in this study with corresponding origin, repository number and reference.

Strain	Origin	DSMZ depository number	Reference
D2^T^	Human skin (country unknown, 1975)	DSM 20326^T^	Kloos and Schleifer (1975)
D3	Clean room, wipe sample (MPS, Göttingen, Germany, 2014)	DSM 31028	
K1	V2A steel (ISS, 2019)	DSM 111179	Sobisch et al. (2019)
H17	Human skin (DLR, Cologne, Germany, 2019)	DSM 113836	

### Cultivation on agar and liquid medium

The *S. capitis* strains were cultivated on solid agar plates for 24 h up to 48 h at 37°C and were further stored at room temperature. In this study, tryptic soy agar (TSA, 17 g/L casein peptone, 3 g/L soy peptone, 5 g/L NaCl, 2.5 g/L K_2_HPO_4_, 2.5 g/L glucose, 15 g/L agar, and pH 7.3), Reasoners 2A agar (R2A, 0.05 g/L yeast extract, 0.05 g/L proteose peptone, 0.05 g/L casamino acids, 0.05 g/L dextrose, 0.05 g/L soluble starch, 0.03 g/L Na-pyruvate, 0.03 g/L K_2_HPO_4_, 0.005 g/L MgSO4, 15 g/L agar, and pH 7.2), and Columbia blood agar (23 g/L peptone, 1 g/L starch, 5 g/L NaCl, 50 ml/L sheep blood, 14 g/L agar, and pH 7.5) were used. For cultivation in liquid medium either tryptic soy broth (TSB) or R2A broth was used. For stress response assays, overnight cultures were prepared by inoculating 20 ml of liquid medium in flasks with multiple colonies of the individual strains. Flasks were incubated for 18 h at 37°C at 200 rpm. After incubation, cells were washed twice with phosphate buffered saline (PBS: 7 g/L Na_2_HPO_4_, 3 g/L KH_2_PO_4_, 4 g/L NaCl, and pH 7.5) by centrifugation at 4,000 × *g*, removing the supernatant and resuspending the pellet in PBS. Number of cells was adjusted in PBS either by measuring the optical density at 600 nm (OD_600nm_) in a microplate reader (*Infinite M200 PRO, Tecan, Männedorf, Switzerland*) or by counting using a Thoma counting chamber.

### Identification of strains

#### Matrix assisted laser desorption ionization time-of-flight mass spectrometry

To verify the strains identity, matrix assisted laser desorption ionization time-of-flight mass spectrometry (MALDI-TOF MS) was performed. For this, few colonies were picked from the strains cultivated at 37°C on Columbia agar (5% sheep blood) and applied on a target. For high resolution imaging quality and protecting instrument integrity, 1 μl of fleXmatrix (*Bruker, Billerica, United States*) was added and left to dry. The target was then placed in the MALDI-TOF MS (*Bruker, Billerica, MA, United States*) for automated analysis.

### Genome sequencing

For Illumina sequencing, highly purified DNA was extracted using the column-based DNeasy UltraClean Microbial Kit (*Qiagen GmbH, Hilden, Germany*) according to the manufacturer’s instructions. The obtained DNA was qualitatively and quantitatively evaluated using a UV/Vis spectrophotometer (*NanoDrop OneC, Thermo Fisher Scientific Inc., Waltham, MA, United States*). Dual-indexed Illumina sequencing libraries were constructed from each sample using the NexteraXT kit (*Illumina, San Diego, CA, United States*), pooled, and sequenced on the Illumina MiSeq platform (*Illumina, San Diego, CA, United States*).

For Nanopore sequencing, DNA was extracted using the Quick-DNA HMW MagBead Kit (*Zymo Research, Irvine, CA, United States*) according to the manufacturer’s instructions and frozen at −20°C. Quality of the isolated DNA was checked for fragment length and purity by capillary gel electrophoresis (*AATI Fragment analyzer, DNF 467 genomic DNA kit, Agilent, Santa Clara, CA, United States*) and UV/Vis spectroscopy. About 1–1.5 μg of each genomic DNA sample was then treated with end repair (*E7546, New England Biolabs, Ipswich, MA, United States*) and FFPE repair enzyme mixes (*M6630, New England Biolabs, Ipswich, MA, United States*) to gain blunt ended 3′dA tailed and 5′ phosphorylated DNA with minimum nick damages. Sequencing libraries were then prepared by ligation of molecular barcodes (*EXP-NBD104, Oxford Nanopore Technologies, Oxford, United Kingdom*) and sequencing adapters (*SQK-LSK109*, *Oxford Nanopore Technologies, Oxford, United Kingdom*). Reaction times for the ligation steps in the protocol were prolonged by factor two to maximize ligation efficiency. Final bead purification of pooled sequencing libraries was performed using long fragment buffer from the SQK-LSK109 kit. A total amount of 450 ng pooled library was then sequenced on a R9.4.1 MinION flowcell (*Oxford Nanopore Technologies, Oxford, United Kingdom*) for 48 h. Basecalling of the raw data was performed in live mode using Guppy 3.0.6 and fast basecalling.

Adapters were removed from long sequencing reads with Porechop 0.2.4.^1^ Long reads were then length filtered (min 1 kb) and downsampled to target 120,000 kb using Filtlong 0.2.1. Raw short sequencing reads were processed with Fastp 0.23.2 (Chen et al., 2018). Long sequencing reads were pre-assembled using Flye 2.9 (Kolmogorov et al., 2019) and hybrid assemblies were conducted with Unicycler 0.4.9 (Wick et al., 2017) using short and long sequencing reads as well as pre-assembled long reads.

### Genome characterization and comparative genomics

Assembled genomes were annotated with Bakta 1.3.3 (Schwengers et al., 2021). Annotated genomes were subsequently imported into EDGAR 3 (Dieckmann et al., 2021) in addition to reference genomes of *S. capitis* subsp. *urealyticus* strain DSM 6717^T^ (GCA_002901925.1) and *S. capitis* subsp. *capitis* strain AYP1020 (GCF_001028645.1). ANI values, neighbor-joining phylogenetic trees, based on the core genome, and VENN diagram were created using EDGAR 3. Comparative circular genome plot was created with mummer2circos^2^ with the type strain D2^T^ as reference. Antibiotic resistance genes were detected using abricate 1.0.0 (Seemann, 2020) using the NCBI AMRFinderPlus (Feldgarden et al., 2019) and CARD databases (Jia et al., 2016).

### Determination of growth

To determine the overall growth of the strains, the bacteria were incubated in a 96-well-plate in 200 μl R2A and TSB, respectively, over a period of 18 h at 37°C. Every 30 min, subsequently to 5 s of orbital shaking, the OD_600nm_ was detected using a microplate reader (*Infinite M200 PRO, Tecan, Männedorf, Switzerland*). The initial OD_600nm_ was adjusted to 0.1 for all strains. The experiment was performed in three biological replicates, of which the average OD_600nm_ and the standard error was calculated.

### Extraction of pigments

For pigment extraction, R2A agar plates were inoculated evenly with 1 ml of a cell suspensions with a cell concentration of 10^7^ CFU/ml. Plates were incubated for 24 h at 37°C. After incubation, biomass from two agar plates per strain was collected with sterile inoculation loops and transferred to sterile tubes with 2 ml H_2_O. 200 μl were used to determine the number of CFU/ml and the remaining suspensions were centrifuged for 10 min at 14,000 × *g*. Supernatants were discarded and pellets were frozen at −20°C. The frozen pellets were thawed at room temperature by adding 2 ml methanol (≥99.9%) and were transferred into Lysing Matrix B tubes (*MP Biomedicals, Irvine, CA, United States*). The samples were treated twice for 40 s in a high-speed benchtop homogenizer (*FastPrep, MP Biomedicals, Irvine, CA, United States*) at 6 m/s. Afterward, the samples were heated at 55°C for 5 min and centrifuged at 14.000 rpm for 10 min. The supernatant was removed and transferred to HPLC vials. For measuring the UV/Vis spectrum of the methanol extracts from the strains, 1 ml of the extracts was transferred into 10 mm UV-cuvettes (*Brand, Wertheim, Germany*). Measurement was performed using a UV/Vis spectrophotometer (*NanoDrop 200C, Thermo Fisher Scientific Inc., Waltham, MA, United States*) with methanol (≥99.9%) as a baseline.

### Metabolism

To determine the metabolic and biochemical profiles of the strains the VITEK®2 system (*bioMérieux, Marcy-l’Etoile, France*) and API® test stripes (API®-test Staph, *bioMérieux, Marcy-l’Etoile, France*) were used. The samples for VITEK®2 were prepared according to instructions from the manufacturer by suspending colonies from a Columbia blood agar plate that was incubated for 24 h at 37°C in 0.85% NaCl to achieve a McFarland value of 0.5. The 5 ml reaction tube filled with the adjusted cell suspension was then placed into a carrier together with the VITEK® 2 GP ID Card (*bioMérieux, Marcy-l’Etoile, France*) into the VITEK®2. API® tests were performed according to the instructions of the manufacturer. The test stripes give information about metabolic reactions *via* color changes in the respective test wells. Beforehand, cells were cultivated on Columbia blood agar for 24 h at 37°C and colonies were suspended in API Staph medium according to a McFarland value of 0.5, which was used for inoculation of the test wells. Test stripes were incubated for 24 h at 37°C before reagents were added and tests were evaluated according to the manual. Results were compared to the API® Staph Identification table (*bioMérieux 2013, Ref 20,500*).

### Fatty acids and polar lipid extraction

For extraction of fatty acids and polar lipids, the *S. capitis* strains D2^T^, D3, H17, and K1 were incubated in 150 ml R2A broth each at 37°C in a biological triplicate. Cells were harvested after reaching an OD_600nm_ of 1.0 (± 0.2). 50 mg of the cell material were prepared as described (Sasser, 1990) and the fatty acid methyl esters analyzed *via* gas chromatography (GC System 8890, Agilent Technologies, Santa Clara, United States) with a mass selective detector (GC/MSD 5977B, Agilent Technologies, Santa Clara, United States) with chromatographic conditions described by Lipski and Altendorf (1997). From the total fatty acid content, a weighted average melting temperature (WAMT) was calculated using the sum of each fatty acid’s melting temperature multiplied by its percentage from the total fatty acid content. Polar lipids were extracted from the remaining cell material and visualized using one- and two-dimensional thin-layer chromatography (TLC) and various staining methods, according to Heidler von Heilborn et al. (2021). For two-dimensional TLC a chloroform/methanol/water solvent (65:25:4 v/v) was used for the first dimension and a chloroform/acetic acid/methanol/water solvent (80:15:12:4 v/v) was used for the second dimension. The latter was also used for the one-dimensional TLC.

### Crystal violet biofilm assay

The semiquantitative crystal violet biofilm assay was performed according to Stepanovic et al., 2000 with modifications. Cell suspensions were prepared as described above and the OD_600nm_ was adjusted to 0.1 in TSB. 200 μl of each cell suspension was transferred in triplicates to a 96-well plate. The plate was incubated for 24 h at 37°C. At the end of the incubation period, the supernatant was carefully discarded, followed by two washing steps with 300 μl PBS each. The plate was left to dry under the laminar flow for 10 min. 200 μl 0.5% crystal violet (*Merck, Darmstadt, Germany*) was added into the wells, and the plate was covered with aluminum foil. After 30 min of incubation at room temperature, the supernatant was carefully discarded. Two washing steps with 300 μl H_2_O each were performed. The wells were then filled with 400 μl ethanol (95%), and the plate was placed on the shaker for 5 min to dissolve the crystal violet stain from the biofilm. The amount of dissolved stain was determined by measuring the absorbance at 570 nm inside a plate reader (*Victor Nivo™, PerkinElmer, Waltham, MA, United States*).

### Testing antibiotic susceptibilities by broth microdilution

Susceptibility was determined thrice by broth microdilution. Susceptibility tests were employed strictly according to the manufacturer’s instruction. From each strain, a bacterial suspension in 0.9% saline solution was prepared. The suspension was adjusted to a McFarland value of between 0.48 and 0.52 using a DensiCHEK plus photometer (*bioMerieux, Marcy-l’Etoile, France*). For each broth microdilution, MIC-Plates Micronaut STAPH (*Merlin, Bornheim, Germany*) were taken. Tests were performed with Mueller–Hinton broth (*Merlin, Bornheim, Germany*) and were visually and independently read by three trained observers. Minimal inhibitory concentrations (MIC) were interpreted according to EUCAST breakpoints 2022 v.12.

### Cellular stress response assays

For all stress response assays, cell suspensions were prepared as described before (Cultivation on agar and liquid medium) and colony forming units per ml (CFU/ml) were determined before and after exposure to the different stress conditions. For this, 10-fold dilution series were prepared in PBS and plated on TSA plates, which were incubated at 37°C up to 48 h before counting the colonies of the induvial dilution steps and calculating CFU/ml. Survival fractions were calculated by dividing the number of CFU/mL after exposure (N) by the initial CFU/ml (N_0_). All experiments were performed in biological triplicates and standard error (SE) was calculated. For irradiation with UV-C and X-rays, the dose which is lethal for 90% of cells (LD_90_) was calculated by linear regression of the survival fraction data.

#### Hydrogen peroxide

For hydrogen peroxide treatment, cell suspensions were prepared as described before and an OD_600nm_ of 0.3 was adjusted in PBS. 2 ml reaction tubes were prepared with 100 μl of H_2_O_2_ (15 and 30%) or 100 μl of H_2_O as a control. 900 μl of the cell suspensions were added into the tubes to achieve final H_2_O_2_ concentrations of 1.5 and 3% and initial cell concentrations in the range of 10^8^ CFU/ml. The treatment was stopped after 30 and 60 min, respectively, using 1 mg/ml catalase dissolved in PBS.

#### X-ray

X-ray irradiation was performed according to Cortesão et al. (2020). Cell suspensions of the strains were prepared as described before. For the irradiation, the cell suspensions were diluted in 1:100 in PBS and 100 μl were transferred into PCR tubes. The closed X-ray system (RS225, Gulmay) was used to irradiate the samples at doses of 50, 100, 250, and 500 Gy. An untreated sample was used as a 0 Gy control.

#### UV radiation at 254 nm

UV radiation at 254 nm was performed according to Cortesão et al. (2020) with slight modifications. Cell suspensions were prepared as described before and diluted 1:100 in PBS. 20 ml of the cell suspensions were transferred to sterile petri dishes (9 cm) which were placed on top of a magnetic stirrer under the filtered UV lamp (*254 nm, VL-6.LC, Vilber, Eberhardzell, Germany*). A sterile stirring bar inside the cell suspension was used to create constant stirring during irradiation. Cells were irradiated with the lid of the petri dish open for time spans according to doses of 10, 50, 100, and 150 J/m^2^. An untreated sample was used as 0 J/m^2^ control. After irradiation, CFU/ml were determined and survival fractions were calculated by dividing the number of CFU/ml after irradiation (N) by the initial CFU/mL (N_0_). The dose which is lethal for 90% of cells (LD_90_) was calculated by linear regression of the survival fraction data.

#### Desiccation

For desiccation experiments, cell suspensions in PBS were prepared as described before. 30 μl of the cell suspensions were pipetted in biological triplicates onto small steel disks (7 mm, V4A) resolving in a final cell count of 10^7^ CFU/disk. The steel disks were either desiccated under normal laboratory atmosphere or inside an anaerobic chamber in forming gas (95% N_2_, 5% H_2_). After 24 h, 7, 14, 21, and 28 days, the metal plates were transferred into tubes with 1 ml PBS and vortexed for 30 s.

### Data analysis

Data were plotted as mean values by using R package ggplot2 3.3.5 (Wickham, 2016) with calculated standard error (SE) as error bars. Statistical analysis (Student’s *t*-test) was performed by using Sigma Plot (Version 13.0, Systat Software) with raw data and two-tailed value of *p* of *p* ≤ 0.05 was considered statistically significant.

## Results

### Identification of strains

All four strains were identified *via* MALDI-TOF MS as *S. capitis* (Supplementary Table 1). The first matched pattern for the subspecies was subsp. *capitis* for D2^T^, D3, and H17. For D3, the second matched pattern was subsp. *urealyticus.* The first matched pattern for K1 was subsp. *urealyticus* and the second subsp. *capitis*. In both cases, both matched patterns have a similarly high score value.

### Genomes and phylogenomic analysis

**Figure 1:**
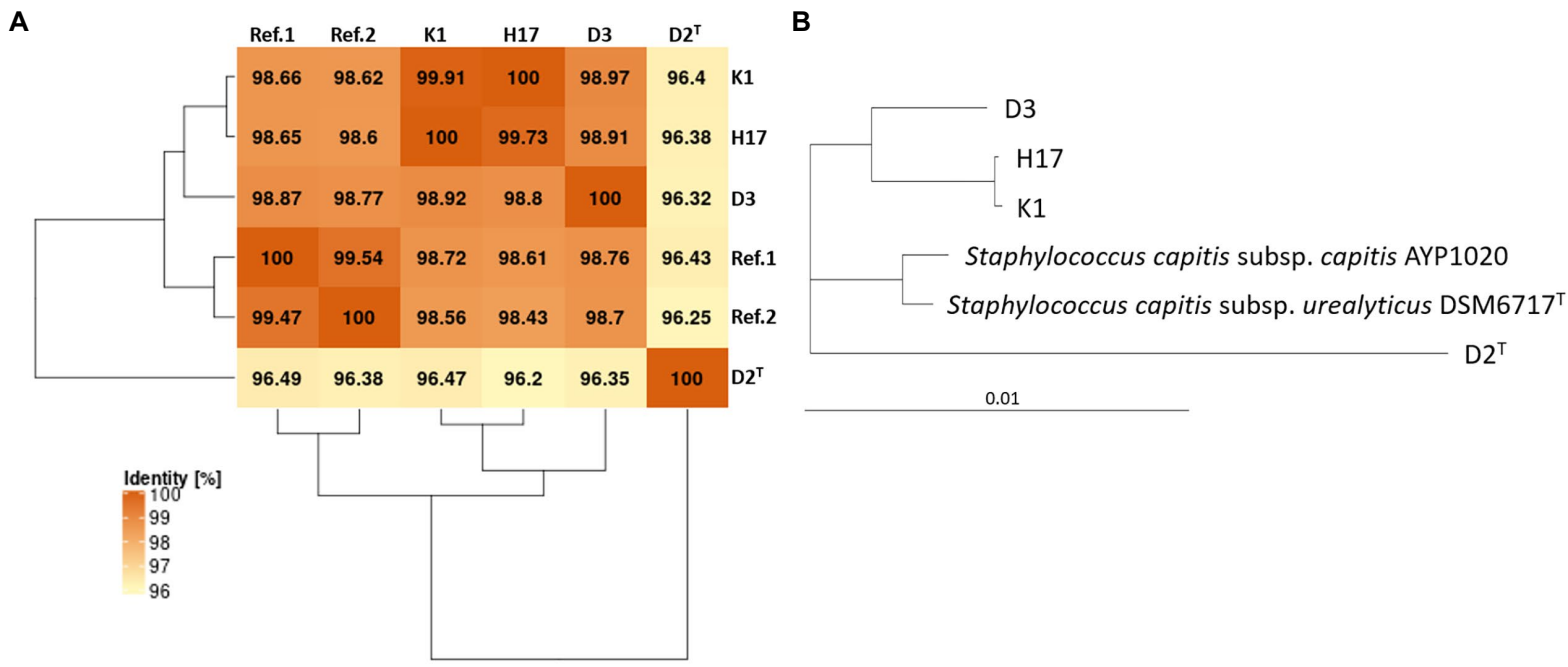
ANI Matrix and phylogenetic tree of *Staphylococcus capitis* strains with reference genome of *Staphylococcus capitis* subsp. *urealyticus* strain DSM 6717^T^ (GCA_002901925.1) and *Staphylococcus capitis* subsp. *capitis* strain AYP1020 (GCF_001028645.1).

Whole genomes of the four strains were sequenced using Illumina MiSeq and Oxford Nanopore Technologies MinION sequencing platforms, respectively. *Via* hybrid assemblies of short and long reads, all sequences of the D2^T^, H17, and K1 isolates were assembled as circular replicons and therefore considered complete. For D3, the chromosome and two additional plasmids were closed; however, seven additional contigs remained (Supplementary Table 2). The length of the chromosomal DNA of all four strains was found to be between 2,400 and 2,500 kb. The analysis of the plasmid showed that D2^T^ and H17 share a plasmid and strains D3 and K1 have a similar plasmid. Mutual average nucleotide identity (ANI) values between the four strains and two reference genomes *S. capitis* subsp. *capitis* strain AYP1020 (GCF_001028645.1) and *S. capitis* subsp. *urealyticus* strain DSM 6717^T^ (GCA_002901925.1), denoted as Ref 1 and Ref 2 in Figure 1A, ranged between 96 and 100% which is above the common species cutoff of 94%. Noticeably, the type strain D2^T^ showed a lower ANI of only 96% against the other strains. In addition, the two reference genomes and the two genomes of K1 and H17 each exhibited high mutual ANI values above 99% (Figure 1A). This close contingency is also depicted in a maximum-likelihood tree that was calculated based on a core genome alignment of the four strains and the two reference strains (Figure 1B).

**Figure 2:**
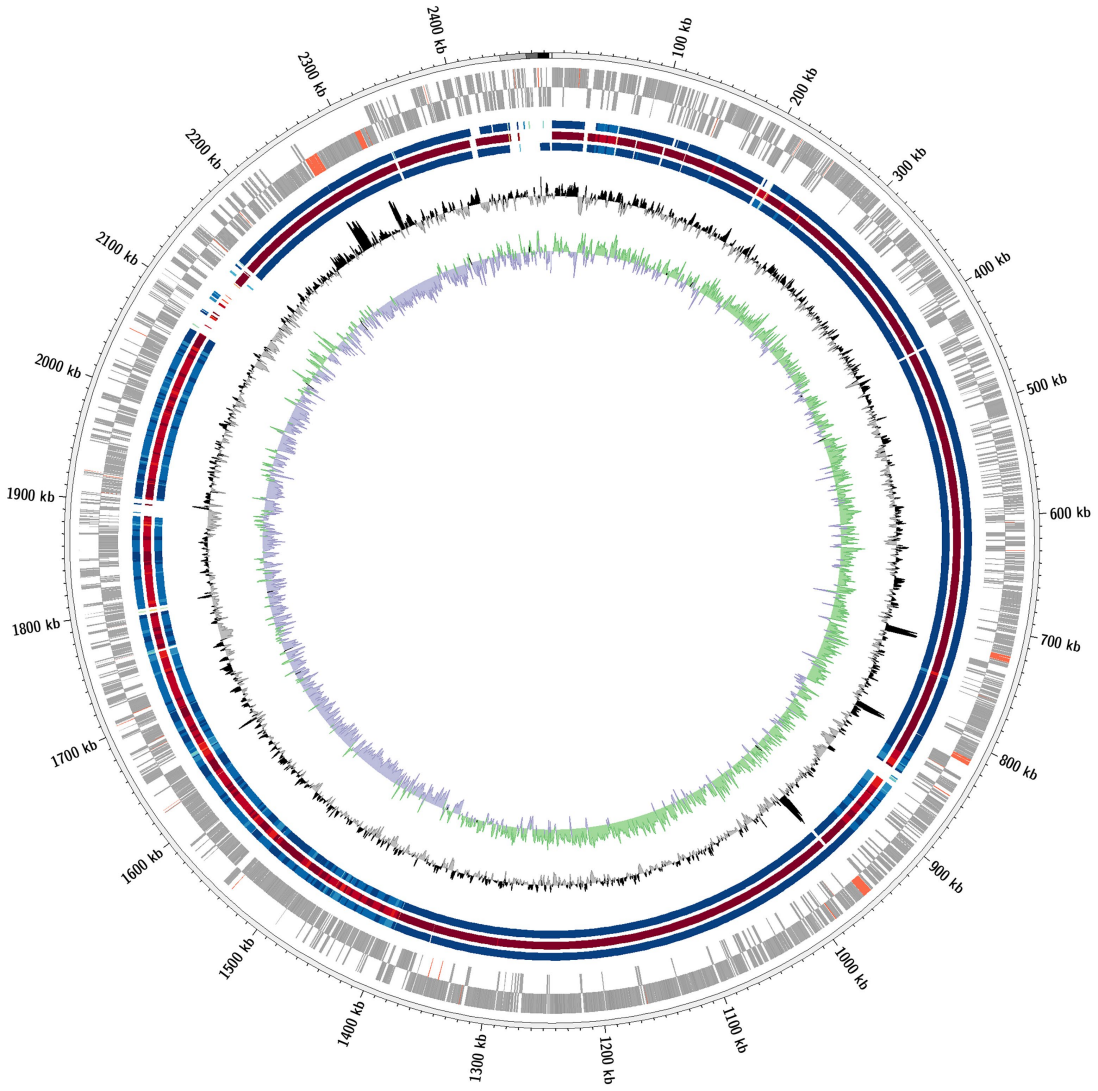
Comparative circular genome plot. Depicted is a comparative circular genome plot of the strains D3, K1 and H17 against the type strain D2^T^. From outward to inward: coding genes (grey) and non-coding genes (red) of the forward and reverse strand, respectively; Homolog genomic regions of strains D3 (blue), K1 (red) and H17 (blue), respectively; GC content and GC skew.

Plotting of the genomes in a circular genome plot (Figure 2) revealed the overall high homology of the genomes of the four strains. Nevertheless, there are parts of the genome that are present in the type strains D2^T^ but are absent in the other three strains. There are 10 smaller areas (<10 kb), two slightly larger areas (10–20 kb), and one large area (80–90 kb) where genes were missing in all three strains (D3, K1, and H17) compared to the type strain (D2^T^).

**Figure 3:**
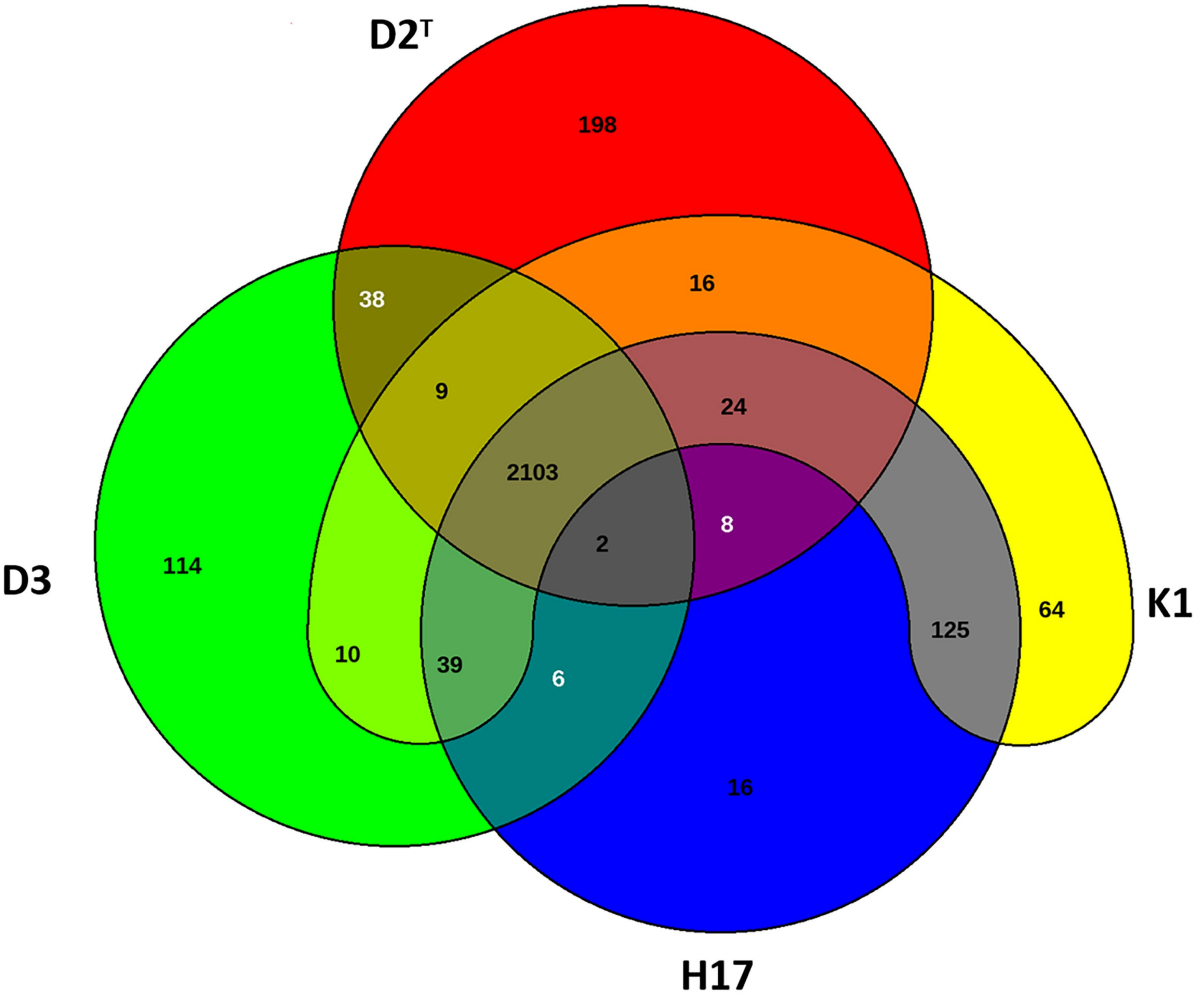
Venn diagram of shared genes and singletons of type strain D2^T^ (red), ISS isolate K1 (yellow), bedrest-study isolate H17 (blue) and clean room isolate D3 (green).

Figure 3 shows the number of reciprocal best hits between genes within the assembled genomes of the four strains. The majority of genes that are considered in the Venn diagram were shared by all four strains (2103). Strain K1, D2^T^, and D3 showed nine orthologous genes that are not present in strain H17. Latter shared two genes with D2^T^ and D3 that were not present in K1. 39 genes were shared by the other three strain but not the type strain D2^T^ and 24 genes were only shared among D2^T^, K1, and H17 and not strain D3. Strain K1 shared 10 genes with strain D3 only, 16 with D2^T^ and 125 genes with H17. The type strain D2^T^ shared 38 genes with strain D3 and eight with H17 whereas strain D3 and H17 shared only six genes. Genes without any hit against the other three genomes, respectively, are listed as singletons in Supplementary Table 3. Among the singletons were many genes encoding for hypothetical proteins but also several encoding for proteins involved in virulence and resistance mechanisms. Further prediction of virulence genes was performed by comparing to virulence genes predicted in *S. capitis* AYP1020 (Cameron et al., 2015) using BLAST. Virulence associated genes with a query coverage and sequence identity of 80% are listed in Supplementary Table 4. Most virulence associated genes that were predicted in AYP1020 were also found in the four strains included in this study. These include *arlRS, rot, sigB, capDACB, hlb, clpP, clpBCX, sepA, htrA, splE, lip, geh1, geh2, lipA, psmα, psmδ, psmβ1a, psmβ1c, psmβ1d, psmβ2, fbe, atlE*, and *ebh*.

### Phenotype

The phenotypes of the four strains were characterized *via* determination of growth, metabolism, fatty acid and polar lipid patterns, biofilm formation as well as susceptibility to antibiotics.

#### Growth

**Figure 4:**
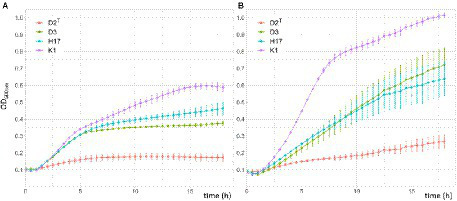
Growth of *S. capitis* strains in double concentrated R2A **(A)** and TSB **(B)** for 18h in a 96-well-plate at 37°C. Measurements were taken in a microplate reader. Initial OD_600nm_ was adjusted to 0.1. Presented OD_600nm_ values are the average of three biological replicates performed in triplicates each. Error bars represent the calculated standard error.

The growth of the four strains was measured *via* the increase in OD_600nm_ over 18 h in R2A broth and TSB inside a 96-well-plate. In both types of media, the lag phase of all four strains was about 1–1.5 h before the bacteria entered the exponential growth phase. In R2A broth, all strains showed a similar growth rate, except D2^T^ which grew considerably slower. The strains D3 and H17 reached the stationary phase after approximately 6 h with still slightly increasing OD_600nm_ of H17. K1 only reached the stationary phase after 15 h, but growth rate was also slowed down after 6 h. In TSB, K1 showed the fastest growth, reaching the highest OD_600nm_ (1.01 after 18 h) of all four strains. At 7.5 h, the growth of K1 slowed down with the OD_600nm_ still slightly increasing until the end of the 18 h incubation period. D3 and H17 showed similar growth rates with increasing OD_600nm_values until the end of the incubation period. Final OD_600nm_ was 0.72 for D3 and 0.64 for H17. As in R2A broth, the type strain D2^T^ showed reduced growth in TSB compared to the other strains and was only reaching an OD_600nm_ of 0.27 after 18 h of incubation (Figure 4).

#### UV/Vis spectra of extracted colony pigments

**Figure 5:**
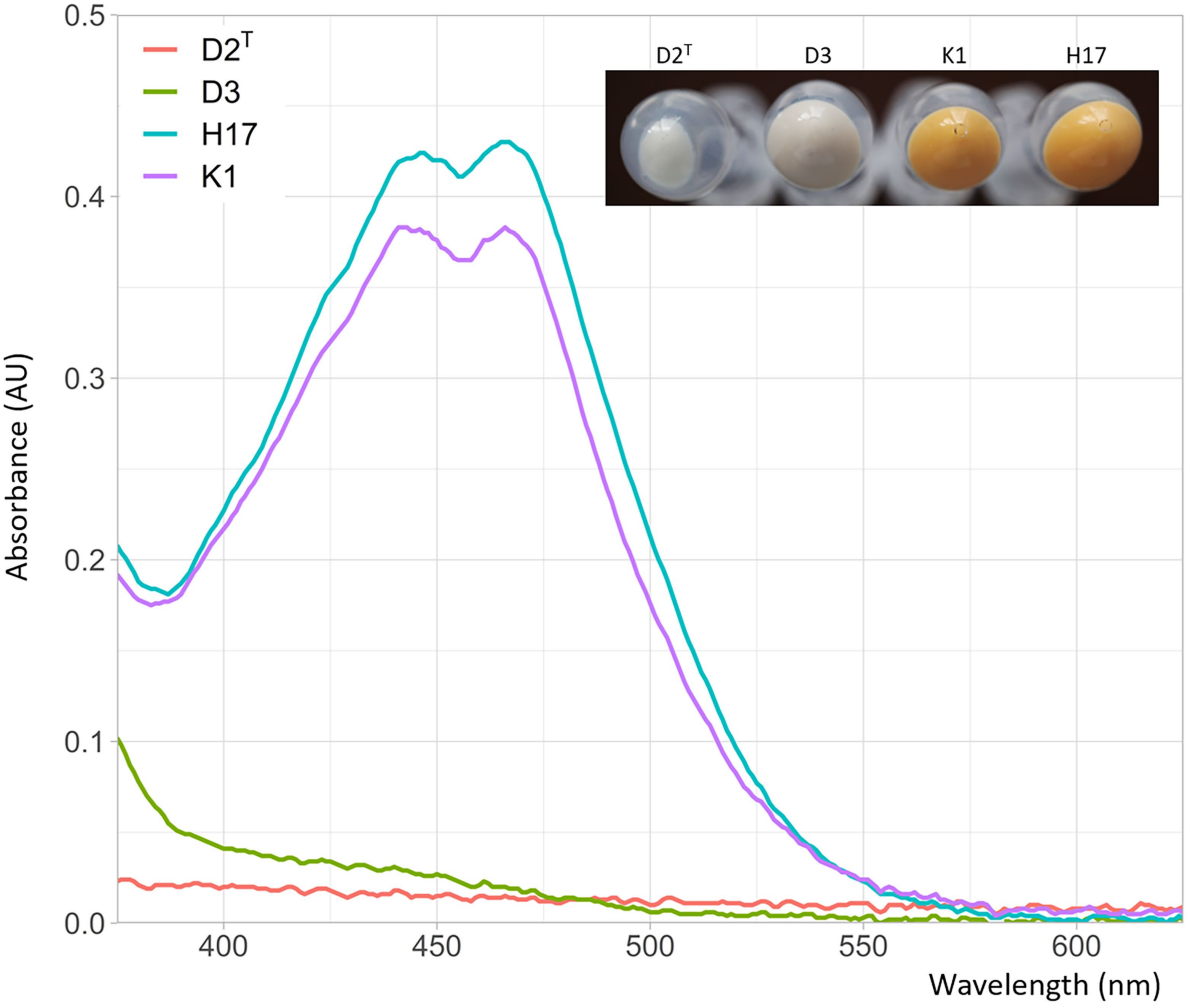
Absorbance of methanol extracts from *Staphylococcus capitis* isolates in absorbance units (AU) at wavelengths of 375–625 nm. Photographic image shows the collected biomass of two inoculated R2A agar plates used for methanol extraction with resulting cell counts of 2.3 x 10^10^ CFU/mL for K1, 4.5 x 10^10^ CFU/mL for H17, 5.0 x 10^8^ CFU/mL for H17 and 5.6 x 10^10^ CFU/mL for D3.

When the strains were cultivated on TSA and R2A agar plates, the strains K1 and H17 exhibited a yellow to orange pigmentation that was neither present in the type strain D2^T^ nor in strain D3. Figure 5 shows the pelleted biomass collected from two R2A agar plates per strain that were incubated for 24 h at 37°C. It is noteworthy that the collectable biomass of type strain D2^T^ was considerably lower than of the other three stains. Figure 5 shows the UV/Vis spectra of the methanol extracts of the cell pellets. K1 and H17 showed two-peak spectra with maximum absorbance at 450 and 466 nm. These peaks were not detected in the two unpigmented strains D2^T^ and D3.

#### Metabolism

Metabolic activity of the four *S. capitis* strains was determined by using the VITEK®2 system and by performing API® tests. Results from the VITEK®2 system are summarized in Supplementary Table 5. It was found that all strains show positive results for arginine dihydrolase, L-pyrrolydonyl-acrylamidase, L-lactate alkalization, growth in 6.5% NaCl, metabolism of D-mannose, and resistance to optochin. The strains differentiated in polymyxin B resistance (K1 positive), metabolism of D-maltose (D3, H17 positive), bacitracin resistance (D2^T^, D3 positive), metabolisms of D-mannitol (D2^T^, K1, and H17 positive), metabolisms of sucrose (K1, H17 positive), and arginine hydrolase activity (D2^T^, K1 positive).

Results of the API® tests are shown in (Table 2). All strains were positive in the metabolism of D-glucose, D-fructose, D-mannose, and production of acetyl-methyl-carbinol (Voges-Proskauer reaction). The strains differentiated in the metabolism of D-maltose (D3, K1, and H17 positive), D-Mannitol (D2^T^, K1, and H17 positive), D-sucrose (D3, K1, and H17 positive), nitrate reaction to nitrite (D2^T^, K1 positive), and alkaline phosphate activity (D3, K1, and H17 positive).

**Table 2:** Test results of all strains in the API® Staph Test after 24 h of incubation at 37°C and addition of reagents.

	D2^T^	D3	K1	H17	Ref.	%
Negative control	−	−	−	−	−	0
Acid formation from D-Glucose	+	+	+	+	+	100
Acid formation from D-Fructose	+	+	+	+	+	99
Acid formation from D-Mannose	+	+	+	+	+	80
Acid formation from D-Maltose	−	+	+	+	−	43
Acid formation from D-Lactose	−	−	−	−	−	22
Acid formation from D-Trehalose	−	−	−	−	−	2
Acid formation from D-Mannitol	+	−	+	+	v	36
Acid formation from D-Xylitol	−	−	−	−	−	0
Acid formation from D-Melibiose	−	−	−	−	−	0
Nitrate reduction to nitrite	+	−	−	+	+	86
Alkaline phosphatase	−	+	+	+	−	23
Acetyl-methyl-carbinol production (Voges-Proskauer)	+	+	+	+	+	90
Acid formation from D-Raffinose	−	−	−	−	−	0
Acid formation from D-Xylose	−	−	−	−	−	0
Acid formation from D-Sucrose	−	+	+	+	- /v	50
Acid formation from Methyl-α-D-Glucopyranoside	−	−	−	−	−	0
Acid formation from N-Acetyl-Glucosamine	−	−	−	−	−	1
Arginine dihydrolase	+	+	+	+	+	85
Urease	−	−	−	−	−	35

Reference (Ref.) strain from manual (Ref. 20,500, Biomerieux) is *S. capitis* subsp. *capitis* strain DSM 6180. Positive reaction is indicated as (+), negative reaction is indicated as (−) and variable reaction is indicated as (v). The outer right column gives the percentage of *S. capitis* strains showing positive reactions according to the manual (Ref. 20,500, Biomerieux) after 18–24 h at 26 ± 2°C.

The API® test results were compared with an identification table (*API® Staph Identification table: bioMérieux 2013, Ref 20,500*). D2^T^ matched the biochemical reaction pattern that most *S. capitis* strains possess, except for D-mannitol metabolism, which is only positive for 36% of all reactions. K1 and H17 were also able to metabolize D-mannitol, leaving D3 as the only strain out of the four that lacked metabolism of D-mannitol. This was confirmed by streaking the strains on mannitol salt phenol red agar (Supplementary Figure 1). The other strains matched the *S. capitis* pattern for the most part, except for the lack of nitrate reduction to nitrite in K1 and H17, which usually is positive in 86% of reactions with *S capitis*. Furthermore, D3, K1, and H17 showed alkaline phosphatase activity, which is only observed in 23% of reactions in *S. capitis* strains and additionally these three strains were able to metabolize D-sucrose, which is according to the identification table only observed in 50% of all *S. capitis* strains.

#### Fatty acids and polar lipids

The analysis of the fatty acid patterns of the four *S. capitis* strains revealed differences while certain fatty acids showed higher variability than others (Table 3). The highest variability was found in the *anteiso* C15:0 fatty acid, which is 9.4% more abundant in the type strain D2^T^ [52.8 (±5.3) %] compared to K1 [43.4 (±0.8) %]. Furthermore, the difference in abundance was about 5% regarding *iso* C19:0 fatty acid between the type strain [1.5 (± 0.5) %] and K1 [6.4 (± 0.4) %] and *anteiso* C19:0 fatty acid between the type strain [4.0 (± 1.3) %] and H17 [9.7 (± 2.7) %]. Noticeably, strain K1 had a higher weighted average melting temperature with 41.8°C compared to the other three strains, which were 38.3°C for strain D2^T^, 38.5°C for D3, and 38.7°C for H17. Furthermore, the results of the two dimensional thin-layer chromatography of the polar lipids (Supplementary Figure 2) revealed the presence of diphosphatidylglycerol, phosphatidylglycerol, and diglucosyldiacylglycerol in all four strains. In strains K1 and D3, lysylphosphatidylglycerol was detected additionally.

**Table 3:** Fatty acid patterns of *Staphylococcus capitis* subsp. *capitis* strains D2^T^, D3, K1 and H17.

Fatty acids	D2^T^	D3	K1	H17
iC14:0	0.5 (± 0.1)	2.4 (± 0.3)	0.5 (± 0.1)	0.5 (± 0.1)
iC15:0	3.5 (± 0.3)	3.6 (± 0.3)	8.1 (± 0.6)	7.1 (± 1.2)
aiC15:0	52.8 (± 5.3)	47.2 (± 4.4)	43.4 (± 0.8)	47.8 (± 5.6)
iC16:0	0.1 (± 0.1)	1.2 (± 0.0)	0.3 (± 0.1)	0.3 (± 0.1)
C16:0	0.6 (± 0.1)	1.0 (± 0.1)	0.2 (± 0.1)	0.1 (± 0.2)
iC17:0	3.4 (± 0.2)	1.9 (± 0.1)	4.7 (± 0.5)	4.8 (± 0.7)
aiC17:0	16.8 (± 0.3)	18.1 (± 0.6)	12.2 (± 1.6)	14.9 (± 0.4)
C18:0	10.0 (± 1.3)	10.7 (± 1.3)	5.9 (± 0.6)	3.9 (± 0.8)
iC19:0	1.5 (± 0.5)	0.9 (± 0.3)	6.4 (± 0.4)	5.3 (± 1.5)
aiC19:0	4.0 (± 1.3)	7.0 (± 1.8)	8.9 (± 1.1)	9.7 (± 2.7)
C20:0	6.7 (± 2.2)	4.7 (± 2.1)	9.1 (± 1.4)	5.2 (± 2.8)
WAMT (°C)	38.3	38.5	41.8	38.7

*Iso* (i) and *anteiso* (ai) fatty acids that are abundant with >1% in at least one of the strains are shown as well as the weighted average melting temperature (WAMT).

#### Biofilm formation

**Figure 6:**
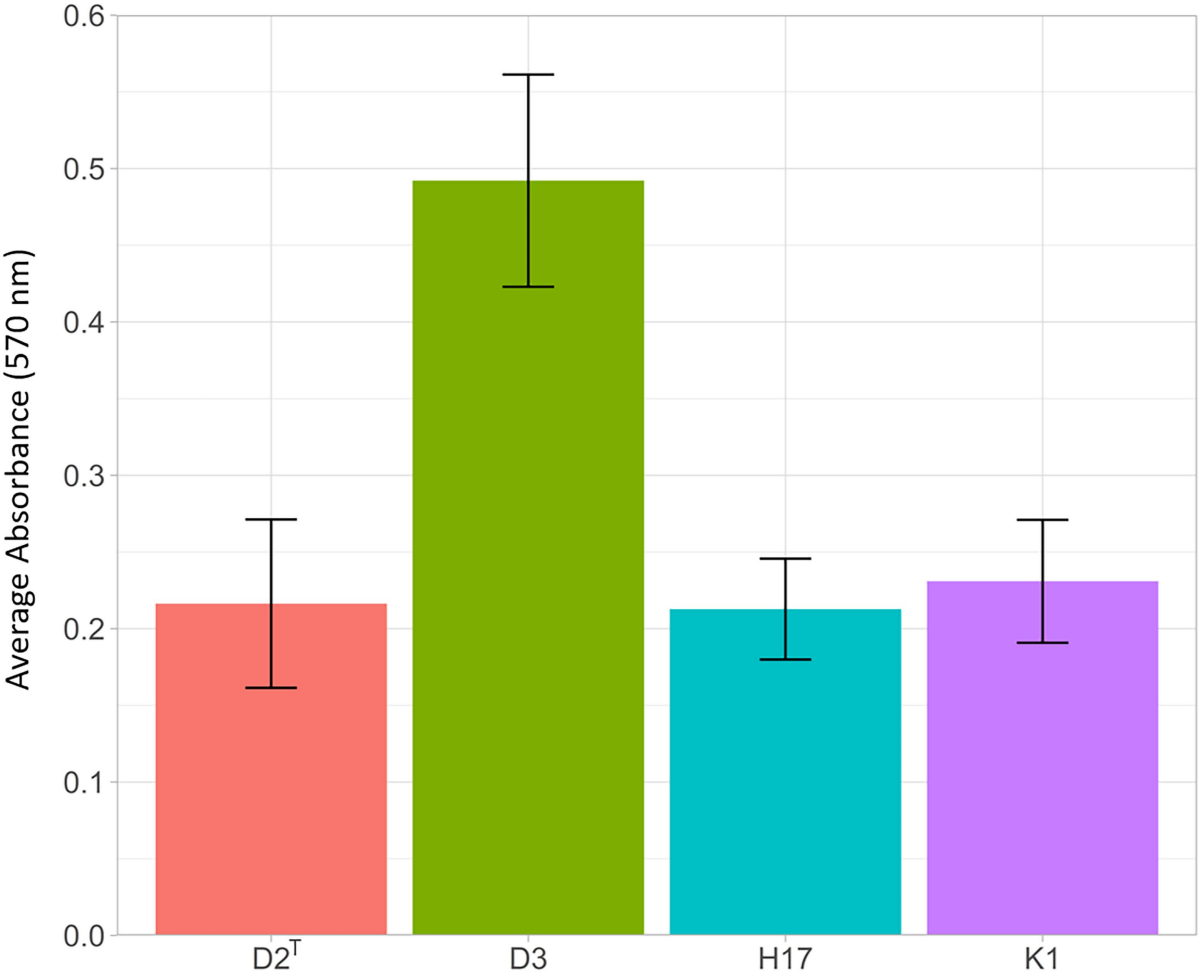
Results of crystal violet biofilm assay. Strains were incubated for 24 h at 37°C in 96-well plates and stained with 0.5% crystal violet for 30 min, after dissolving with ethanol (95%), staining intensity was measured at 570 nm in a 1:10 dilution in PBS. The bars show the average absorbance at 570 nm of three individual experiments with the according standard error.

Biofilm formation was tested by using the semiquantitative crystal violet biofilm assay. The results show increased absorbance at 570 nm for strain D3, which indicates stronger biofilm formation in this strain compared to the other three strains (Figure 6). However, this result is not statistically significant (*t*-test D2^T^/D3: *p* = 0,129).

#### Susceptibility to antibiotics

Susceptibility to antibiotics was tested *via* broth microdilution. According to the EUCAST breakpoints 2022 v.12., the four strains are considered susceptible to all tested antibiotics except fosfomycin (Table 4). Here, the MIC was 64 mg/L or higher for strains D2^T^, D3, and K1. For strain H17, the MIC was 32 mg/L. For all other tested antibiotics, the MICs were similar among the four tested strains. Additionally, the AMRFinderPlus tool was used to search the assembled genomes for resistance genes. Here, only in strain D2^T^, the *fosB* gene could be detected that is encoding the fosfomycin resistance bacillithiol transferase (Supplementary Table 6). Within the genomes of the strains, further genes regarding resistance against ß-lactam antibiotics were detected in D2^T^ (*blaTEM*) as well as in K1 and H17 (*blaI, blaR1,* and *blaZ*; Supplementary Table 6). Expression of these resistance genes could not be confirmed in this study, since only clinically relevant β-lactamase-stable penicillins (Oxacillin) and cephalosporins (Ceftaroline, Cefoxitin) were tested in the broth microdilution.

**Table 4:** Susceptibility to antibiotics determined *via* broth microdilution.

Strain	Ceftaroline	Cefoxitin	Daptomycin	Erythromycin	Fosfomycin	Fusidic acid	Gentamicin	Gentamicin (high level)	Linezolid	Moxifloxacin	Oxacillin	Rifampicin	Synercid	Trimethoprim/ Sulfamethoxazole	Tigecycline	Teicoplanin	Vancomycin
D2^T^	≤0.25	≤2	≤0.5	=1	>64	≤1	≤0.5	≤128	=2	≤0.25	≤0.125	≤0.0625	≤0.5	=0.5/ 9.5	=0.25	≤0.125	=0.5
D3	≤0.25	≤2	=0.5/1	=1	>64	≤1	≤0.5	≤128	=2	≤0.25	≤0.125/ =0.25	≤0.0625	≤0.5	=0.5/ 9.5	=0.25/ 0.5	=0.5	=0.5
K1	≤0.25	≤2/ =4	≤0.5/ =1	=0.5/ 1	=64	≤1	≤0.5	≤128	=2	≤0.25	≤0.125	≤0.0625	≤0.5	=0.5/ 9.5	=0.25	≤0.125	=0.5/1
H17	≤0.25	≤2/ =4	≤0.5	=0.5/1	=32	≤1	≤0.5	≤128	=2	≤0.25	=0.25	≤0.0625	≤0.5	=0.5/ 9.5	=0.25	≤0.125/ =0.25	=0.5

MIC values are shown in mg/L.

### Stress response

**Figure 7:**
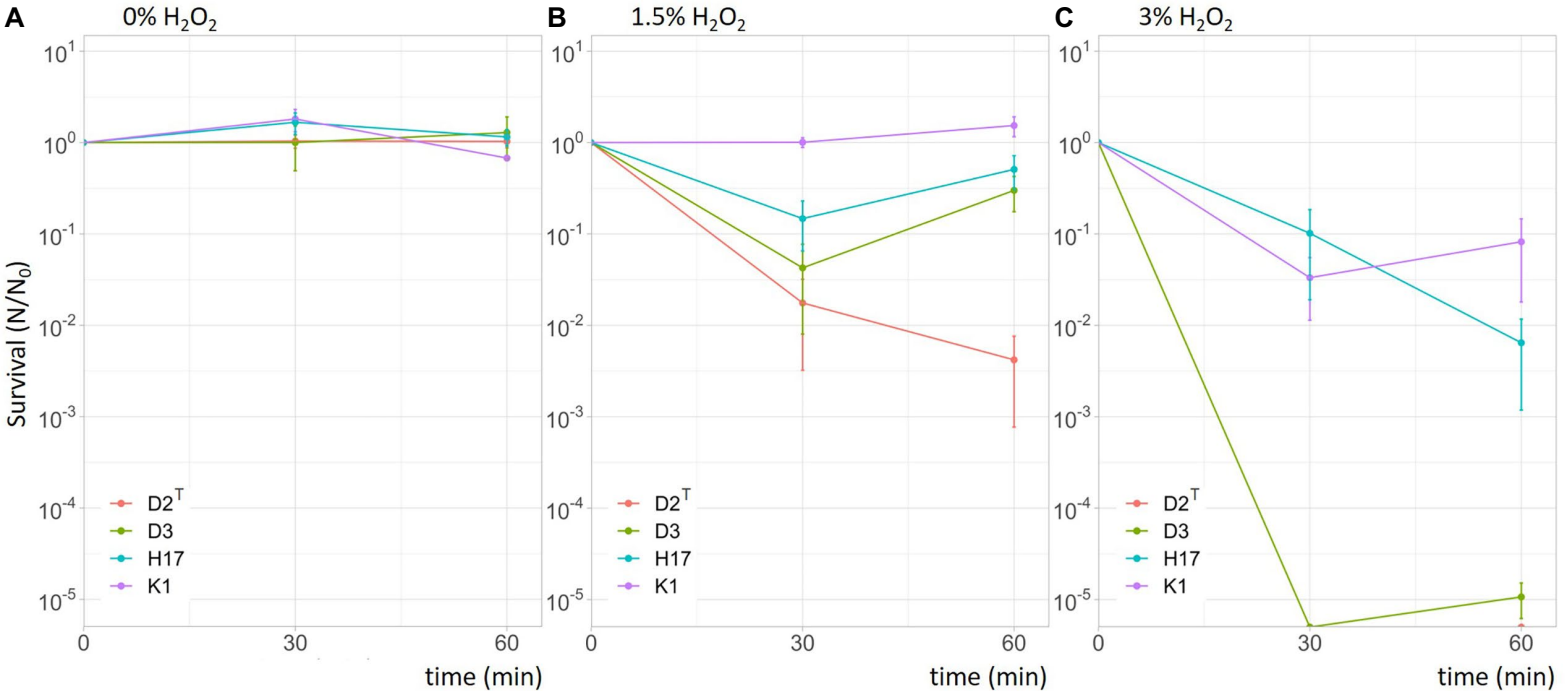
Survival of *S. capitis* strains in 0% **(A)**, 1.5% **(B)** and 3% **(C)** hydrogen peroxide for 30- and 60-min. Experiment was performed with an initial OD_600nm_ of 0.3 resulting in initial cell counts (0 min) of 10^8^ CFU/mL. The experiment was performed in three biological replicates. Error bars represent the calculated standard error.

The results of the stress response assays are summarized in Table 5. The strains were tested for their survival in 1.5 and 3% H_2_O_2_ for 0, 30, and 60 min and in 0% as a control. The survival fraction did not change within the 60 min incubation period inside the untreated control (Figure 7A). The treatment with 1.5% H_2_O_2_ led to a decrease in the survival fraction in D2^T^, D3, and H17 after 30 min, whereby only in D2^T^, survival decreased further at 60 min treatment (Figure 7B) Treatment with 3% H_2_O_2_ led to a decline in the survival fraction of all four strains but to different extends. After 30 min of treatment, the number of CFU of the strains D2^T^ and D3 were below the detection limit (40 CFU/ml), whereas K1 and H17 were reduced only by one order of magnitude (Figure 7C) with K1 showing a significant higher survival compared to the type strain. After 60 min of treatment with 3% H_2_O_2_, the survival fraction of K1 was still about 10^−1^ and the survival fraction of H17 decreased to 10^−2^_._ Survival of D2^T^ could still not be detected since the number of CFU/mL was below the detection limit. The survival fraction of D3 was still low (10^−5^) but in contrast to the 30 min treatment, detectable.

**Table 5:** Survival of *S. capitis* strains in different stress conditions.

Stress condition	D2^T^	D3	K1	H17	Reference
Survival fraction after 60 min treatment with 1.5% H_2_O_2_	4.2 × 10^−3^ (σ 5.9 × 10^−3^)	3.0 × 10^−1^ (σ 2.2 × 10^−1^)	1.5 × 10^0^ (σ 6.5 × 10^−1^)*	3.6 × 10^−1^ (σ 3.6 × 10^−1^)	Figure 7B
Survival fraction after 60 min treatment with in 3% H_2_O_2_	below detection limit	1.1 × 10^−5^ (σ 7.8 × 10^−6^)	8.2 × 10^−2^ (σ 1.1 × 10^−1^)	6.5 × 10^−3^ (σ 9.1 × 10^−3^)	Figure 7C
LD_90_ (X-ray)	96.3 Gy (σ 23.0 Gy)	120.8 Gy (σ 21.5 Gy)	97.6 Gy (σ 13.7 Gy)	89.3 Gy (σ 6.6 Gy)	Figure 8A
LD_90_ (UV-C)	26.2 J/m^2^ (σ 1.9 J/m^2^)	36.4 J/m^2^ (σ 14.5 J/m^2^)	22.3 J/m^2^ (σ 3.5 J/m^2^)	56.6 J/m^2^ (σ 38.5 J/m^2^)	Figure 8B
Survival fraction after 28 days of desiccation (aerobe)	4.2 × 10^−7^ (σ 2.9 × 10^−7^)	4 × 10^−3^ (σ 1.8 × 10^−3^)*	1.9 × 10^−5^ (σ 1.6 × 10^−5^)	6.9 × 10^−4^ (σ 8.1 × 10^−4^)	Figure 9A
Survival fraction after 28 days of desiccation (forming gas)	1.1 × 10^−5^ (σ 6.62 × 10^−6^)	2.5 × 10^−2^ (σ 1.3 × 10^−2^)*	1.4 × 10^−3^ (σ 1.3 × 10^−3^)	1.7 × 10^−2^ (σ 1.6 × 10^−4^)	Figure 9B

For irradiation experiments LD_90_ was calculated *via* linear regression. For other stress response assays survival fraction was calculated as average of biological triplicates with according standard deviation (σ). *T*-tests were performed to determine statistical differences in stress response of the strains compared to the type strain D2^T^. Statistically significant difference (*p* ≤ 0.05) is indicated by (*).

**Figure 8:**
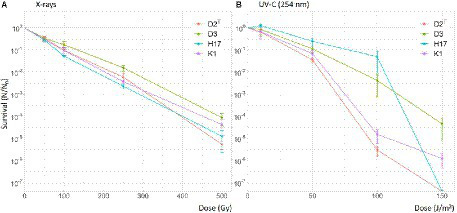
Survival of *S. capitis* during X-ray and UV-C irradiation in PBS. Initial cell counts used for irradiation were 10^7^ CFU/mL. Irradiation experiments were performed in three biological replicates. Error bars represent the calculated standard error.

Furthermore, the strains were tested for their survival during exposure to X-ray irradiation up to a dose of 500 Gy. Calculated LD_90_ values are presented in Table 5. The survival curves of the four strains look very similar (Figure 8A). This is also leading to similar LD_90_ values, which are highest in strain D3 with 128 Gy and lowest in H17 with 90.3 Gy. In regard to irradiation with UV-C, the strains also showed similar survival curves up to a dose of 50 J/m^2^ and then leading to a higher survival fraction of the strains H17 and D3 at 100 J/m^2^ (Figure 8B). However, at a dose of 150 J/m^2^, no CFU were detectable for the strains H17 and D2^T^. Leaving a survival fraction of 10^−4^ for D3 and 10^−6^ for K1. Calculated LD_90_ values were in the range of 25.7 J/m^2^ for D2^T^ up to 55.1 J/m^2^ for H17.

**Figure 9:**
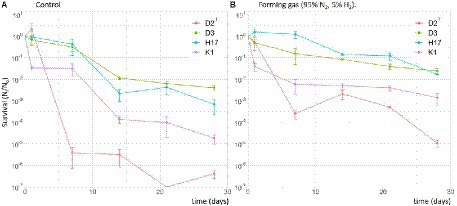
Survival of *S. capitis* strains during desiccation for up to 28 days in normal atmospheric conditions in the laboratory as control **(A)** and in forming gas inside an anaerobic chamber **(B)**. Initial cell counts were 10^7^ CFU/mL. Experiment was performed in biological triplicates. Error bars represent the calculated standard error.

In the desiccation assay, all strains showed a considerably different survival depending on the desiccation conditions. The survival of all strains was higher in forming gas inside the anaerobic chamber compared to normal atmospheric conditions in the lab (Figure 9). The survival fraction of all four strains declined over the time of 28 days but, for all strains CFU could still be recovered after 28 days. Strain D2^T^ seemed to be most susceptible to desiccation in general, leading to lower survival fraction values. Most resistant to desiccation were the strains D3 and H17 which showed a similar survival rate over the whole 28 days of desiccation. However, only D3 showed a significant higher survival compared to the type strain.

## Discussion

### Genotype and phylogeny

Some studies have reported that spaceflight conditions lead to increased virulence in bacteria for example in *Salmonella typhimurium* (Wilson et al., 2007) and *Serratia marcescens* (Gilbert et al., 2020). Other studies reported increased biofilm formation (Kim et al., 2013; Mora et al., 2019) and antibiotic resistances (Fajardo-Cavazos and Nicholson, 2016). In this study, we were aiming for a detailed and systematic comparison of a *S. capitis* subsp. *capitis* space flight isolate (K1), the DSMZ type strain (D2^T^) and two other space flight relevant strains (bedrest study isolate H17, clean room isolate D3) to investigate possible phenotypic and specific genomic differences among these strains. All four strains could be classified as *S. capitis* and belong most likely to the subspecies *capitis* since one of the key features of the other subspecies *S. capitis* subsp. *urealyticus* is positive urease activity (Bannermann and Kloos, 1991) and all four strains showed negative urease activity in both systems tested (VITEK and API). On a phylogenomic level, the type strain DSM 20326^T^ seems to be the most distant related to the other strains including also the available reference genome of strain AYP1020 and of the *S. capitis* subsp. *urealyticus* type strain DSM 6717^T^. In this study, we present the closed genomes of the type strain as well as of the strains K1 and H17. Only the genome of strain D3 could not be closed. Nevertheless, all strains share the same genome size about 2,400–2,500 kb with approximately 2,300 encoded genes, which is according to other already sequenced *S. capitis* genomes. Most genes are shared by all four strains and we could not find additional virulence genes in one strain compared to the others. However, most of the virulence genes that were predicted before in *S. capitis* by Cameron et al. (2015) were also detected in all four strains in this study including several global regulators such as *sigΒ* or *agrADCB,* the biofilm formation associated *ica* operon and genes encoding for exoenzymes and proinflammatory peptides.

### Growth

Looking at phenotypic differences, the ISS isolate K1 is the strain that grows fastest of all four strains in R2A broth and in TSB. Mauclaire and Egli (2010) have observed faster growth of *Micrococcus luteus* space and Earth isolates under simulated microgravity conditions back on Earth. However, no faster growth of the space isolates in normal Earth gravity was reported, only faster growth in simulated microgravity could be detected. The same observation was made in *Pseudomonas aeruginosa* cultured in microgravity (Kim et al., 2013) and *Escherichia coli* cultured in low-shear modeled microgravity (Kim et al., 2014). Furthermore, a 60-fold increase in biomass was observed in *Vibrio natriegens* after cultivation in a 2-D clinostat at 60 rpm (Garschagen et al., 2019).

### Fatty acids and polar lipids

The fatty acid pattern of the four strains mostly matches the composition previously described for *S. capitis* (Durham and Kloos, 1978), however, there seems to be a high variability among the four strains in this study especially regarding the C15 and C19 fatty acids. A previous study could not detect a difference of the fatty acid composition in pigmented and non-pigmented *Staphylococcus aureus* (*S. aureus*; Tiwari et al., 2018). In our study, the most prominent differences in the fatty acid patterns of the pigmented strains compared to the non-pigmented strains were a higher presence of *iso* C19:0 fatty acids and a lower presence of C18:0. Interestingly, the ISS isolate K1 has a higher weighted average melting temperature (WAMT) based on the fatty acid composition compared to the other three strains. Under varying environmental conditions such as different temperatures, bacteria adjust the lipid composition of the cell membrane (Zhang and Rock, 2008). With decreasing growth temperatures, bacteria form shorter and more unsaturated and branched-chain fatty acids, which result in a lower WAMT (Suutari and Laakso, 1994). Since *S. capitis* is associated with the human skin (Byrd et al., 2018), the source of this strain was probably an astronaut of the ISS crew present during the time of the exposure experiment (Sobisch et al., 2019). A study found that the human’s core body temperature is gradually increasing during spaceflight about 1°C in total (Stahn et al., 2017). This increase in body temperature might also influence associated skin microbiome leading for example to changes in the fatty acid composition and WAMT. Regarding polar lipids, all four strains contained diphosphatidylglycerol, phosphatidylglycerol and diglucosyldiacylglycerol but only strains K1 and D3 contained lysylphosphatidylglycerol, which is a considerable difference within the same species.

### Susceptibility to antibiotics

In this study, we could not detect a difference in antibiotic susceptibility between the ISS isolate and the three other strains. According to the EUCAST breakpoints 2022 v.12., the strains were only considered resistant to fosfomycin. A previous study by Fajardo-Cavazos and Nicholson (2016), showed that the cultivation of *S. epidermidis* on the ISS led to changes in the spectrum of mutations in the *rpoB* gene leading to rifampicin resistance. In this study, no changes in neither the susceptibility to rifampicin nor any other tested antibiotic were detected. The genotypes could not exactly predict the actual expressed resistances in the four strains, since only in strain D2^T^ *fosB* was detected. The expression of the other detected resistance genes (*blaTEM*, *blaI, blaR1,* and *blaZ*) could not be confirmed in this study, since only β-lactamase-stable penicillin (Oxacillin) and cephalosporins (Ceftaroline, Cefoxitin) that have clinical relevance were tested in the broth microdilution.

### Pigmentation and response to stress conditions

The most prominent phenotypic difference between the four strains is that the strains K1 and H17 produce an orange pigment when grown on TSA or R2A agar which is neither observed in the type strain nor in strain D3. *S. capitis* subsp. *capitis* was first described as unpigmented (Bannerman and Kloos, 1991) but 73% of *S. capitis* subsp. *urealyticus* strains show delayed yellow pigmentation. In the UV/Vis spectra, the methanol extracts of the two pigmented strains show the typical absorbance spectrum of carotenoids solved in methanol (Zang et al., 1997). As integral parts of membranes or cell walls, pigments can offer protection against environmental conditions such as cold temperatures, UV-radiation or oxidative stress, which is for example induced during exposure to hydrogen peroxide (Arrage et al., 1993; Dieser et al., 2010; Mohana et al., 2013; Seel et al., 2020).

In this study, we focused on the stress factors regarding the spacecraft indoor environment, including stressors such as desiccation, decontamination measures (H_2_O_2_ and UV-C irradiation) as well as X-rays as a reference for ionizing radiation. Strains K1, H17 and D3 show increased resistance to desiccation compared to the type strain. It was observed before that pigmented strains of *S. aureus* are less susceptible to desiccation (Beard-Pegler et al., 1988) and most staphylococci isolated from clinical sources show pigmentation which is lost after incubation *in vitro* (Grinsted and Lacey, 1973). In contrast to that, this study showed that the unpigmented strain D3 also shows a high resistance to desiccation, indicating that other factors apart pigmentation influence desiccation tolerance. One factor could be that this strain was isolated within a clean room facility since it has been shown before that clean room conditions can favor more resistant bacteria in regards to tolerance to desiccation, UV-C radiation as well as resistance to hydrogen peroxide (La Duc et al., 2003). This is especially relevant in spacecraft assembly facilities, where strict decontamination protocols are followed to avoid forward contamination of other celestial bodies. In this context, van Heereveld et al. (2017), have used *Staphylococcus xylosus* as model organism for forward contamination to Mars from clean room facilities and found a high survival potential.

Furthermore, it was observed that all four strains survived desiccation in forming gas conditions better than under normal atmospheric conditions. In strain K1, this effect was observed before when the strain was desiccated in a Mars-analogue gas on a scientific balloon mission to the stratosphere (Cortesão et al., 2021). Desiccation induces stress through the presence of different reactive oxygen species (ROS; França et al., 2007). It was shown before that the presence of oxygen is decreasing bacterial survival under desiccating conditions (Potts, 1994; Beblo-Vranesevic et al., 2017). Looking further at the resistance of the four strains to ROS it was observed that the two pigmented *S. capitis* strains K1 and H17 showed better survival after H_2_O_2_ treatment compared to the two unpigmented strains. This could be due to the known antioxidant properties of carotenoids such as staphyloxanthin, which can scavenge radicals through their conjugated double bonds (El-Agamey et al., 2004; Clauditz et al., 2006). In *S. aureus,* the golden pigment staphyloxanthin is an important virulence factor due to its antioxidative activity which protects cells from immune responses such as neutrophil killing (Liu et al., 2005). In contrast to this, no difference in the susceptibility to UV-C nor X-ray irradiation was observed in this study in between the pigmented and non-pigmented strains. It was shown before that staphyloxanthin-deficient *S. aureus* (*crt*-mutants) are not more sensitive to X-rays, however, these mutants showed threefold increased sensitivity to UV-C (Pannu et al., 2019). Based on our finding here, future studies would also benefit from testing further stress factors that are more relevant to outer space conditions. This would include, amongst other methods, testing the survival of *S. capitis* after exposure to cosmic rays, vacuum UV and polychromatic UV radiation as well as other atmospheric conditions such vacuum or Mars atmosphere. For strain K1, this was partly tested in the previously mentioned scientific balloon mission to the stratosphere (Cortesão et al., 2021). In this study, *S. capitis* strain K1 was able to survive desiccated in simulated Mars atmosphere for 5 months shielded from UV radiation. However, no survival could be detected in the samples that were exposed to UV.

### Biofilm formation

The *icaADBC* operon has been linked to biofilm formation in coagulase negative staphylococci (Heilmann et al., 1996; de Silva et al., 2002). D3, K1, and H17 encode the complete *ica* operon in their genome whereas the type strain D2^T^ lacks one gene of the *ica* operon namely *icaD,* which encodes for the poly-beta-1,6-N-acetyl-D-glucosamine synthesis protein IcaD, important for slime production in *S. aureus* (Arciola et al., 2001). In the semiquantitative crystal violet biofilm assay, the type strain D2^T^ showed no difference regarding biofilm formation compared to the ISS isolate K1 or the bedrest-study isolate H17. Here, strain D3, the clean room isolate, shows the highest biofilm formation compared to the other three strains. Although this assay has clear limitations, it can give robust indication on a strains tendency to form biofilms. The ability to form strong biofilms and its high desiccation tolerance might make strain D3 a very robust strain that can even survive the unfavorable conditions of a clean room facility as already mentioned in the previous section. In general, biofilm formation is one of the most important virulence factors of coagulase-negative staphylococci such as *Staphylococcus capitis* (Cui et al., 2013), and space conditions were reported to increase tendencies toward biofilm formation and surface interactions (Mora et al., 2019). On this regard it is worth noting that the ISS isolate is able to form biofilms, however, based on our data, no significant increase in biofilm formation can be observed.

## Conclusion

Concluding the results of this in-depth comparison of a *S. capitis* spaceflight isolate to the type strain and two other spaceflight relevant strains, lead to the discovery of certain differences among the strains, regarding growth, colony pigmentation, biofilm formation, fatty acid composition, polar lipids as well as resistance toward desiccation and H_2_O_2_. Due to the small sample size of only comparing one isolate from the ISS and three from Earth, only careful conclusions on the effect of space conditions on phenotype and genotype of *S. capitis* can be drawn. Moreover, the *S. capitis* type strain DSM 20326^T^ appears to be degenerated particularly compared to the other strains as it shows slow growth and low tolerance to desiccation, which confounds a comprehensive comparison between the different strains. Generally speaking, the observed differences between the strains are not necessarily indicating an increased virulence of the spaceflight isolate. Nevertheless. the occurrence of pigmented strains in spaceflight associated environments should be studied in more detail. Particularly, since pigmentation is involved in the tolerance to ROS which was confirmed in this study by the tolerance of the two pigmented strains to H_2_O_2_ treatment. Increased resistance to H_2_O_2_ is especially concerning since the ISS surfaces are cleaned with H_2_O_2_ wipes for disinfection. Furthermore, this study presented, that a prediction of the bacterial phenotype *via* the genotype is in some cases not sufficient if the actual threat to astronaut health during spaceflight is supposed to be evaluated. This was especially shown in the prediction of antibiotic resistances. Therefore, we support a more systematic characterization of spaceflight isolates for example from the ISS. This should include next to bacteria also other microorganisms such as filamentous fungi and viruses. Tracking the phenotypic characteristics of the current ISS microbiome would benefit the actual hazard assessment of microbial contamination for crew and equipment. This would accordingly support finding appropriate countermeasures against harmful microbial species in the spaceflight indoor environment without over- or underestimating the actual safety risk for the crew. Additionally, we think that *S. capitis* as human-associated bacterium is a valuable model organism for further astrobiological studies focusing on topics such as limits of life, lithopanspermia and planetary protection.

## Data availability statement

The data presented in the study are deposited in the GenBank repository. Accession numbers can be found in the Supplementary material (Supplementary Table 2).

## Author contributions

KS, AR, DH, OS, LK, FA, CN, and JM performed the experiments and subsequent data analysis. KS, AR, OS, DH, CN, JM, and FA were involved in manuscript preparation. KR, MP, AL, TH, and RM contributed to the methodological design of the study and the preparation of the manuscript. All authors contributed to the article and approved the submitted version.

## Funding

KS and RM were supported by the DLR grant FuE-Projekt “ISS LIFE” (Programm RF-FuW, Teilprogramm 475) and are part of the ESA ISS project: “Testing antimicrobial metal surfaces under spaceflight conditions—an effective strategy to prevent microbial biofilm formation” [No. ESA-HSO-ESR-ILSRA-2014-054; Biofilm Inhibition on Flight equipment and on board the ISS using microbiologically Lethal Metal Surfaces (BIOFILMS)] and the NASA-ESA-DLR joint bed rest study AGBRESA (Artificial Gravity Bed Rest Study). KR and RM were supported by ESA grant “BioProtect—Bioinspired Shielding Material for Radiation Protection Purposes” (ESA Contract No. 4000137602/22/NL/GLC/my).

## Conflict of interest

The authors declare that the research was conducted in the absence of any commercial or financial relationships that could be construed as a potential conflict of interest.

## Publisher’s note

All claims expressed in this article are solely those of the authors and do not necessarily represent those of their affiliated organizations, or those of the publisher, the editors and the reviewers. Any product that may be evaluated in this article, or claim that may be made by its manufacturer, is not guaranteed or endorsed by the publisher.

## Footnotes

^1^ https://github.com/rrwick/Porechop.git

^2^ https://github.com/metagenlab/mummer2circos
