# Supplementary material for: Phenotypic and genomic assessment of the potential threat of human spaceflight-relevant Staphylococcus capitis isolates under stress conditions
Source: Front Microbiol. 2022 Nov 3;13:1007143. doi: 10.3389/fmicb.2022.1007143 (PMC9669719; doi:10.3389/fmicb.2022.1007143)
Supplement: Supplementary file 1 [file Data_Sheet_1.docx]

Supplementary Material

# MALDI-TOF MS identification of strains

Table 1 Results of MALDI-TOF MS identification of strains. Table shows the top two results for each strain with the according matched pattern, score and rank value.

| **Strain** | **Matched pattern** | **Score value** | **Rank value** |
| --- | --- | --- | --- |
| D2^T^ | *Staphylococcus capitis* ssp *capitis* DSM 20326^T^ | 2.27 | 1 (+++) |
|  | *Staphylococcus capitis* ssp *capitis* DSM 6180 | 2.12 | 2 (+++) |
| D3 | *Staphylococcus capitis* ssp *capitis* DSM 20326^T^ | 2.05 | 1 (+++) |
|  | *Staphylococcus capitis* ssp *urealyticus* DSM 6718^T^ | 2.01 | 2 (+++) |
| K1 | *Staphylococcus capitis* ssp *urealyticus* DSM 6718^T^ | 2.22 | 1 (+++) |
|  | *Staphylococcus capitis* ssp *capitis* DSM 20326^T^ | 2.17 | 2 (+++) |
| H17 | *Staphylococcus capitis* ssp *capitis* DSM 20325 | 2.11 | 1 (+++) |
|  | *Staphylococcus capitis* ssp *capitis* DSM 20326^T^ | 2.11 | 2 (+++) |

# Genome Assembly

Table 2 Results of genome assembly of the four *S. capitis* strains D2^T^, D3, K1 and H17. Shared plasmids among the strains are indicated in the same color.

| **D2^T^** | | | | |
| --- | --- | --- | --- | --- |
| **Contig** | **Length (bp)** | **Depth** | **Circular** | **Accession number** |
| 1 | 2,443,707 | 1.00x | yes | CP103049.1 |
| 2 | 21,427 | 2.40x | yes | CP103050.1 |
| 3 | 9,586 | 0.26x | yes | CP103051.1 |
| 4 | 9,275 | 7.41x | yes | CP103052.1 |
| 5 | 2,329 | 30.75x | yes | CP103053.1 |
| **D3** | | | | |
| 1 | 2,413,010 | 1.00x | yes | JANTNV000000000 |
| 2 | 21,159 | 2.32x | yes |  |
| 3 | 9,278 | 1.78x | no |  |
| 4 | 8,138 | 0.39x | no |  |
| 5 | 4,460 | 2.25x | yes |  |
| 6 | 3,717 | 0.35x | no |  |
| 7 | 3,099 | 0.34x | no |  |
| 8 | 3,095 | 0.48x | no |  |
| 9 | 1,803 | 1.15x | no |  |
| 10 | 1,666 | 0.66x | no |  |
| **K1** | | | | |
| 1 | 2,517,449 | 1.00x | yes | CP102087.1 |
| 2 | 29,363 | 1.69x | yes | CP102088.1 |
| **H17** | | | | |
| 1 | 2,482,563 | 1.00x | yes | CP102089.1 |
| 2 | 9,274 | 8.49x | yes | CP102090.1 |

# Singletons

Table 3 Singleton genes and according encoded proteins of the four *S. capitis* strains D2^T^, D3, K1 and H17 predicted by EDGAR 3.0.

| **D2^T^** | |
| --- | --- |
| **Singleton:** | **Protein:** |
| NF399_12420 | ABC transporter permease |
| NF399_00245 | ABC-type transport system involved in multi-copper enzyme maturation, permease component |
| NF399_10505 | Alkylmercury lyase family protein |
| NF399_10685 | Antitoxin |
| NF399_00890 | Aspartate transcarbamylase |
| NF399_10625 | ATP-binding protein |
| NF399_09480 | Bacteriocin (Lactococcin_972) |
| NF399_09475 | Bacteriocin-associated integral membrane protein |
| NF399_04325 | Bacteriophage resistance protein |
| NF399_12205 | Chitosanase |
| NF399_10640 | Chitosanase |
| NF399_10620 | DDE-type integrase/transposase/recombinase |
| NF399_00560 | DJ-1/PfpI family protein |
| NF399_02075 | DNA (cytosine-5-)-methyltransferase |
| NF399_00565 | DUF2992 domain-containing protein |
| NF399_11730 | DUF3139 domain-containing protein |
| NF399_10800 | DUF334 domain-containing protein |
| NF399_04390 | DUF4865 domain-containing protein |
| NF399_10660 | Endoglucanase C |
| NF399_10635 | Endoglucanase C307 |
| NF399_12200 | Endoglucanase E1 |
| NF399_12395 | Endoribonuclease MazF |
| NF399_09515 | GRAM-POS-ANCHORING domain-containing protein |
| NF399_04300 | HTH cro/C1-type domain-containing protein |
| NF399_04305 | HTH-17 domain-containing protein |
| NF399_10400 | hypothetical protein |
| NF399_00950 | hypothetical protein |
| NF399_10670 | hypothetical protein |
| NF399_00895 | hypothetical protein |
| NF399_10700 | hypothetical protein |
| NF399_01130 | hypothetical protein |
| NF399_12400 | hypothetical protein |
| NF399_09450 | hypothetical protein |
| NF399_12460 | hypothetical protein |
| NF399_01125 | hypothetical protein |
| NF399_12490 | hypothetical protein |
| NF399_12510 | hypothetical protein |
| NF399_09465 | hypothetical protein |
| NF399_04320 | hypothetical protein |
| NF399_00130 | hypothetical protein |
| NF399_10675 | hypothetical protein |
| NF399_10435 | hypothetical protein |
| NF399_10485 | hypothetical protein |
| NF399_04400 | hypothetical protein |
| NF399_09620 | hypothetical protein |
| NF399_10290 | hypothetical protein |
| NF399_00140 | hypothetical protein |
| NF399_12470 | hypothetical protein |
| NF399_04380 | hypothetical protein |
| NF399_10415 | hypothetical protein |
| NF399_04375 | hypothetical protein |
| NF399_10445 | hypothetical protein |
| NF399_01010 | hypothetical protein |
| NF399_04310 | hypothetical protein |
| NF399_04385 | hypothetical protein |
| NF399_10455 | hypothetical protein |
| NF399_03845 | hypothetical protein |
| NF399_11745 | hypothetical protein |
| NF399_10705 | hypothetical protein |
| NF399_09525 | hypothetical protein |
| NF399_10450 | hypothetical protein |
| NF399_09460 | hypothetical protein |
| NF399_09520 | hypothetical protein |
| NF399_00135 | hypothetical protein |
| NF399_12530 | hypothetical protein |
| NF399_12515 | hypothetical protein |
| NF399_12485 | hypothetical protein |
| NF399_12455 | hypothetical protein |
| NF399_00905 | hypothetical protein |
| NF399_04355 | hypothetical protein |
| NF399_10530 | hypothetical protein |
| NF399_04295 | Lambda repressor-like protein |
| NF399_04395 | LysR-family regulatory protein |
| NF399_12495 | Membrane protein |
| NF399_08975 | Membrane protein |
| NF399_10785 | MobC domain-containing protein |
| NF399_10790 | Mobilization protein |
| NF399_08950 | Oligopeptide ABC transporter |
| NF399_04315 | Pathogenicity island protein |
| NF399_04335 | Pathogenicity island protein |
| NF399_04330 | Prim-Pol domain-containing protein |
| NF399_10580 | Putative dehydrogenases |
| NF399_04290 | Putative integrase |
| NF399_00900 | Putative molybdopterin biosynthesis protein moeB |
| NF399_10680 | Putative mRNA interferase YoeB |
| NF399_12465 | Putative transposase |
| NF399_12585 | Replication protein |
| NF399_10465 | Ribosomal-protein-alanine acetyltransferase |
| NF399_10795 | RLX protein |
| NF399_09505 | Transcriptional regulator |
| NF399_12415 | Transcriptional regulator, TetR family |
| NF399_00935 | Type I restriction modification DNA specificity protein |
| **D3** | |
| **Singleton:** | **Protein:** |
| NF397_00295 | AAA family ATPase |
| NF397_06980 | ABC transporter permease |
| NF397_11835 | ABC transporter, ATP-binding protein |
| NF397_00305 | AlwI family type II restriction endonuclease |
| NF397_00340 | ATP-dependent helicase |
| NF397_00205 | Dihydroneopterin aldolase |
| NF397_00770 | DUF2089 domain-containing protein |
| NF397_09560 | HTH cro/C1-type domain-containing protein |
| NF397_09555 | HTH cro/C1-type domain-containing protein |
| NF397_09530 | hypothetical protein |
| NF397_09535 | hypothetical protein |
| NF397_11975 | hypothetical protein |
| NF397_07280 | hypothetical protein |
| NF397_09465 | hypothetical protein |
| NF397_00745 | hypothetical protein |
| NF397_00325 | hypothetical protein |
| NF397_09540 | hypothetical protein |
| NF397_07275 | hypothetical protein |
| NF397_00970 | hypothetical protein |
| NF397_12150 | hypothetical protein |
| NF397_00250 | hypothetical protein |
| NF397_00755 | hypothetical protein |
| NF397_00310 | hypothetical protein |
| NF397_00705 | hypothetical protein |
| NF397_00715 | hypothetical protein |
| NF397_12070 | hypothetical protein |
| NF397_00135 | hypothetical protein |
| NF397_00290 | hypothetical protein |
| NF397_07255 | hypothetical protein |
| NF397_00330 | hypothetical protein |
| NF397_12115 | hypothetical protein |
| NF397_00740 | hypothetical protein |
| NF397_00315 | hypothetical protein |
| NF397_00720 | hypothetical protein |
| NF397_01200 | hypothetical protein |
| NF397_00765 | hypothetical protein |
| NF397_00320 | hypothetical protein |
| NF397_11795 | hypothetical protein |
| NF397_07260 | hypothetical protein |
| NF397_09460 | hypothetical protein |
| NF397_07265 | hypothetical protein |
| NF397_00285 | hypothetical protein |
| NF397_09565 | Integrase |
| NF397_09525 | Mobile element-associated protein |
| NF397_12175 | Mobilization protein |
| NF397_00225 | Multicopper oxidase mco |
| NF397_09520 | Nucleoside triphosphatase |
| NF397_12165 | OrfE |
| NF397_00335 | ParB N-terminal domain-containing protein |
| NF397_09515 | Pathogenicity island protein |
| NF397_09545 | Pathogenicity island protein |
| NF397_09550 | Pathogenicity island protein |
| NF397_09485 | Pathogenicity island protein |
| NF397_00200 | persulfide dioxygenase-sulfurtransferase CstB |
| NF397_00760 | PH domain-containing protein |
| NF397_07270 | Phage minor structural protein GP20 |
| NF397_00195 | putative protein ORF C17 |
| NF397_09500 | SaPI1 ORF5-like protein |
| NF397_09480 | Sigma70-r4 domain-containing protein |
| NF397_00300 | Site-specific DNA-methyltransferase (adenine-specific) |
| NF397_00130 | Site-specific DNA-methyltransferase (adenine-specific) |
| NF397_02955 | Symporter |
| NF397_12170 | tetracycline efflux MFS transporter Tet(K) |
| NF397_09470 | Transcriptional regulator |
| NF397_00165 | VanY domain-containing protein |
| **K1** | |
| **Singleton:** | **Protein:** |
| NF398_00300 | 3-dmu-9-3-mt domain-containing protein |
| NF398_00255 | AAA-31 domain-containing protein |
| NF398_00260 | Antitoxin MazE |
| NF398_00135 | CPBP family intramembrane metalloprotease |
| NF398_00130 | HTH cro/C1-type domain-containing protein |
| NF398_00185 | hypothetical protein |
| NF398_00200 | hypothetical protein |
| NF398_00240 | hypothetical protein |
| NF398_00320 | hypothetical protein |
| NF398_12460 | hypothetical protein |
| NF398_00210 | hypothetical protein |
| NF398_00270 | hypothetical protein |
| NF398_00265 | hypothetical protein |
| NF398_00330 | hypothetical protein |
| NF398_00190 | hypothetical protein |
| NF398_00390 | hypothetical protein |
| NF398_00145 | hypothetical protein |
| NF398_00250 | hypothetical protein |
| NF398_00315 | hypothetical protein |
| NF398_00165 | Major facilitator superfamily permease |
| NF398_00285 | MFS domain-containing protein |
| NF398_00325 | MobL relaxases |
| NF398_00195 | POLAc domain-containing protein |
| NF398_00175 | Putative DNA binding 3-demethylubiquinone-9 3-methyltransferase domain protein |
| NF398_00420 | Replication initiator protein |
| NF398_00205 | SF3 helicase domain-containing protein |
| NF398_00180 | Transcriptional regulator |
| NF398_00305 | Transcriptional regulator |
| NF398_00160 | Transcriptional regulator, LysR |
| NF398_00405 | Truncated transposase |
| NF398_00400 | Truncated transposase |
| NF398_00410 | Truncated transposase |
| **H17** | |
| **Singleton:** | **Protein:** |
| NF392_00200 | ATP-binding protein |
| NF392_00215 | hypothetical protein |
| NF392_00170 | hypothetical protein |
| NF392_00205 | hypothetical protein |
| NF392_00165 | hypothetical protein |
| NF392_00175 | hypothetical protein |
| NF392_00230 | Mur-ligase-M domain-containing protein |
| NF392_00220 | Putative glycosyl transferase |
| NF392_00130 | Restriction endonuclease subunit R |
| NF392_00135 | Type I restriction-modification system S subunit |

# Virulence genes

Table 4 Prediction of virulence genes in the four *S. capitis* strains D2^T^, D3, K1 and H17 via comparison to *S. capitis* AYP1020 virulence genes according Cameron et al. 2015 using BLAST. Genes were considered as present if query coverage and sequence identity were above 80%.

| **Function** | **Genes** | **Product** | **Locus *S. capitis* AYP1020** | **D2^T^** | **D3** | **K1** | **H17** |
| --- | --- | --- | --- | --- | --- | --- | --- |
| **Global regulators** | agrADCB | Accessory gene regulator | 1223-6 | yes  yes  no  yes | no  yes  yes  yes | no  yes  no  yes | no  yes  no  yes |
|  | sarARVZ | Staphylococcal accessory regulator | 2371  1469  1442  1576 | no  no  no  no | no  no  no  no | no  no  no  no | no  no  no  no |
|  | saeRS | SaeRS two-component regulatory system | 0073–4 | yes  no | yes  yes | yes  yes | yes  yes |
|  | arlRS | ArlRS two-component regulatory system | 0725–6 | yes  yes | yes  yes | yes  yes | yes  yes |
|  | rot | Repressor of toxins | 1043 | yes | yes | yes | yes |
|  | sigB | RNA polymerase sigma factor B | 1257 | yes | yes | yes | yes |
| **Biofilm/PNAG** | icaRADBC | Intercellular adhesion proteins | 1906–1910 | yes  yes  yes  no  yes | yes  yes  yes  yes  yes | yes  yes  yes  yes  yes | yes  yes  yes  yes  yes |
| **PGA** | capDACB | capDACB | 1692  1694–6 | yes  /  yes  yes | yes  yes  yes | yes  yes  yes | yes  yes  yes |
| **Exoenyzmes** | hlb | Beta Hemolysin  Hemolysin III  Hemolysin | 1985  1362 | yes  / | yes  / | yes | yes |
|  | clpP | Clp protease, proteolytic subunit | 0135 | yes | yes | yes | yes |
|  | clpBCX | Clp protease, ATP binding subunits | 0244  2273  0957 | yes  yes  yes | yes  yes  yes | yes  yes  yes | yes  yes  yes |
|  | sepA | Metalloprotease elastase  Zinc metalloprotease  Serine protease, putative | 1844  0571  0279 | yes  yes  yes | yes  yes  yes | yes  yes  yes | yes  yes  yes |
|  | htrA | HtrA like protease, putative | 1007 | yes | yes | yes | yes |
|  | splE | Serine protease  Serine protease | 1979  2166 | yes  yes | yes  yes | yes  yes | yes  yes |
|  | lip | Lipase | 1943 | yes | yes | yes | yes |
|  | geh1 | Lipase | 1911 | yes | yes | yes | yes |
|  | geh2 | Lipase | 2083 | yes | yes | yes | yes |
|  | lipA | Lipase/esterase  Esterase  Esterase | 0022  1534  1701 | yes  yes  yes | yes  yes  yes | yes  yes  yes | yes  yes  yes |
|  | psmα | Phenol Soluble modulin alpha | 2169 | yes | yes | yes | yes |
| **Pro-inflammatory Peptides** | psmδ | Phenol Soluble modulin delta | 2168 | yes | yes | yes | yes |
|  | psmβ1a | Phenol Soluble modulin beta 1a | 0480 | yes* | yes | yes | yes |
|  | psmβ1c | Phenol Soluble modulin beta 1b | 0481 | yes | yes | yes | yes |
|  | psmβ1d | Phenol Soluble modulin beta 1c | 0482 | yes | yes | yes | yes |
|  | psmβ2 | Phenol Soluble modulin beta 2 | 0479 | yes | yes | yes | yes |
|  | hld | Delta Hemolysin | 1222 | no | yes | yes | yes |
|  | fbe | fibronectin binding protein | 0515 | yes | yes | yes | yes |
| **Surface proteins/adhesins** | atlE | Bifunctional autolysin | 0374 | yes | yes | yes | yes |
|  | pls | Plasmin-sensitive surface protein | 1833 | no | no | no | no |
|  | aap | Accumulation associated protein | 2027 | no | no | no | yes |
|  | ebh | Cell wall associated fibronectin binding protein (ECM-binding protein-like protein) | 0751 | yes | yes | yes | yes |

# VITEK

Table 5 Biochemical analysis of *S. capitis* strains D2^T^, D3, K1 and H17 by VITEK® 2 GP ID Card. Positive reaction is indicated with “+” and green filling, negative reaction is indicated with “-“ and red filling.

|  | **D2^T^** | **D3** | **K1** | **H17** |
| --- | --- | --- | --- | --- |
| D-AMYGDALIN | **-** | **-** | **-** | **-** |
| PHOSPHATIDYLINOSITOL PHOSPHOLIPASE C | **-** | **-** | **-** | **-** |
| D-XYLOSE | **-** | **-** | **-** | **-** |
| ARGININE DIHYDROLASE 1 | **+** | **+** | **+** | **+** |
| BETA-GALACTOSIDASE | **-** | **-** | **-** | **-** |
| ALPHA-GLUCOSIDASE | **-** | **-** | **-** | **-** |
| Ala-Phe-Pro ARYLAMIDASE | **-** | **-** | **-** | **-** |
| CYCLODEXTRIN | **-** | **-** | **-** | **-** |
| L-Aspartate ARYLAMIDASE | **-** | **-** | **-** | **-** |
| BETA GALACTOPYRANOSIDASE | **-** | **-** | **-** | **-** |
| ALPHA-MANNOSIDASE | **-** | **-** | **-** | **-** |
| PHOSPHATASE | **-** | **-** | **-** | **-** |
| Leucine ARYLAMIDASE | **-** | **-** | **-** | **-** |
| L-Proline ARYLAMIDASE | **-** | **-** | **-** | **-** |
| BETA GLUCURONIDASE | **-** | **-** | **-** | **-** |
| ALPHA-GALACTOSIDASE | **-** | **-** | **-** | **-** |
| L-Pyrrolydonyl-ARYLAMIDASE | **+** | **+** | **+** | **+** |
| BETA-GLUCURONIDASE | **-** | **-** | **-** | **-** |
| Alanine ARYLAMIDASE | **-** | **-** | **-** | **-** |
| Tyrosine ARYLAMIDASE | **-** | **-** | **-** | **-** |
| D-SORBITOL | **-** | **-** | **-** | **-** |
| UREASE | **-** | **-** | **-** | **-** |
| POLYMIXIN B RESISTANCE | **-** | **-** | **+** | **-** |
| D-GALACTOSE | **-** | **-** | **-** | **-** |
| D-RIBOSE | **-** | **-** | **-** | **-** |
| L-LACTATE alkalization | **+** | **+** | **+** | **+** |
| LACTOSE | **-** | **-** | **-** | **-** |
| N-ACETYL-D-GLUCOSAMINE | **-** | **-** | **-** | **-** |
| D-MALTOSE | **-** | **+** | **-** | **+** |
| BACITRACIN RESISTANCE | **+** | **+** | **-** | **-** |
| NOVOBIOCIN RESISTANCE | **-** | **-** | **-** | **-** |
| GROWTH IN 6.5% NaCl | **+** | **+** | **+** | **+** |
| D-MANNITOL | **+** | **-** | **+** | **+** |
| D-MANNOSE | **+** | **+** | **+** | **+** |
| METHYL-B-D-GLUCOPYRANOSIDE | **-** | **-** | **-** | **-** |
| PULLULAN | **-** | **-** | **-** | **-** |
| D-RAFFINOSE | **-** | **-** | **-** | **-** |
| O/129 RESISTANCE (comp.vibrio.) | **+** | **+** | **+** | **+** |
| SALICIN | **-** | **-** | **-** | **-** |
| SACCHAROSE/SUCROSE | **-** | **-** | **+** | **+** |
| D-TREHALOSE | **-** | **-** | **-** | **-** |
| ARGININE DIHYDROLASE 2 | **+** | **-** | **+** | **-** |
| OPTOCHIN RESISTANCE | **+** | **+** | **+** | **+** |

# Growth on Mannitol Salt Phenol Red Agar


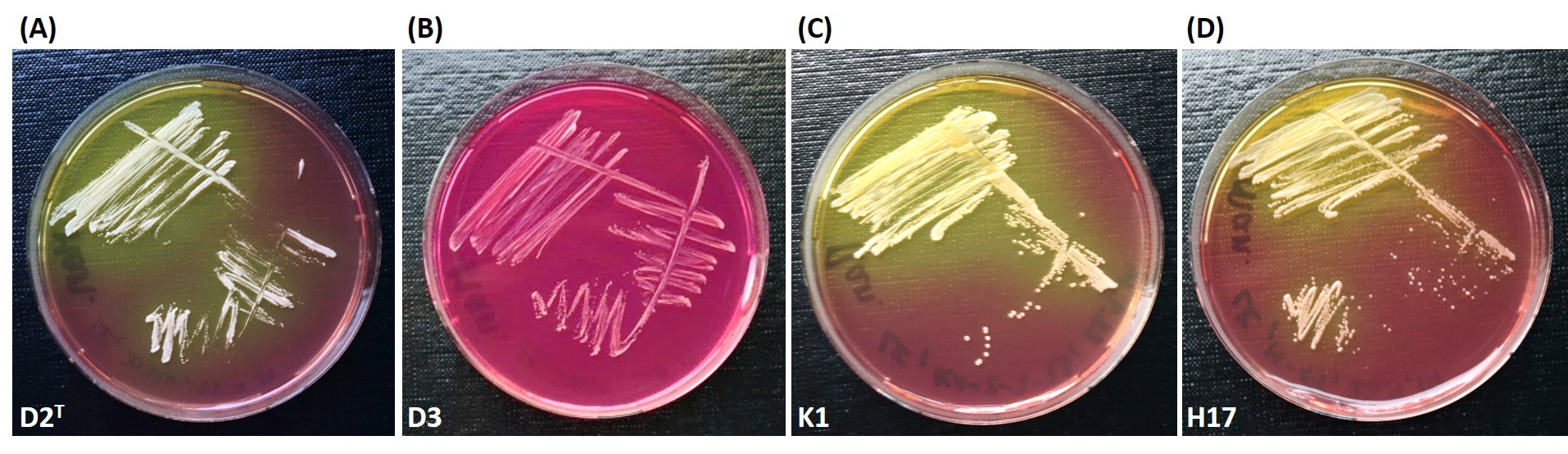


Figure 1 *S. capitis* strains D2^T^ (A), D3 (B), K1 (C) and H17 (D) on mannitol salt phenol red agar after incubation for 48 h at 37 °C. Yellow coloration of the agar indicates metabolism of mannitol.

# Polar lipids


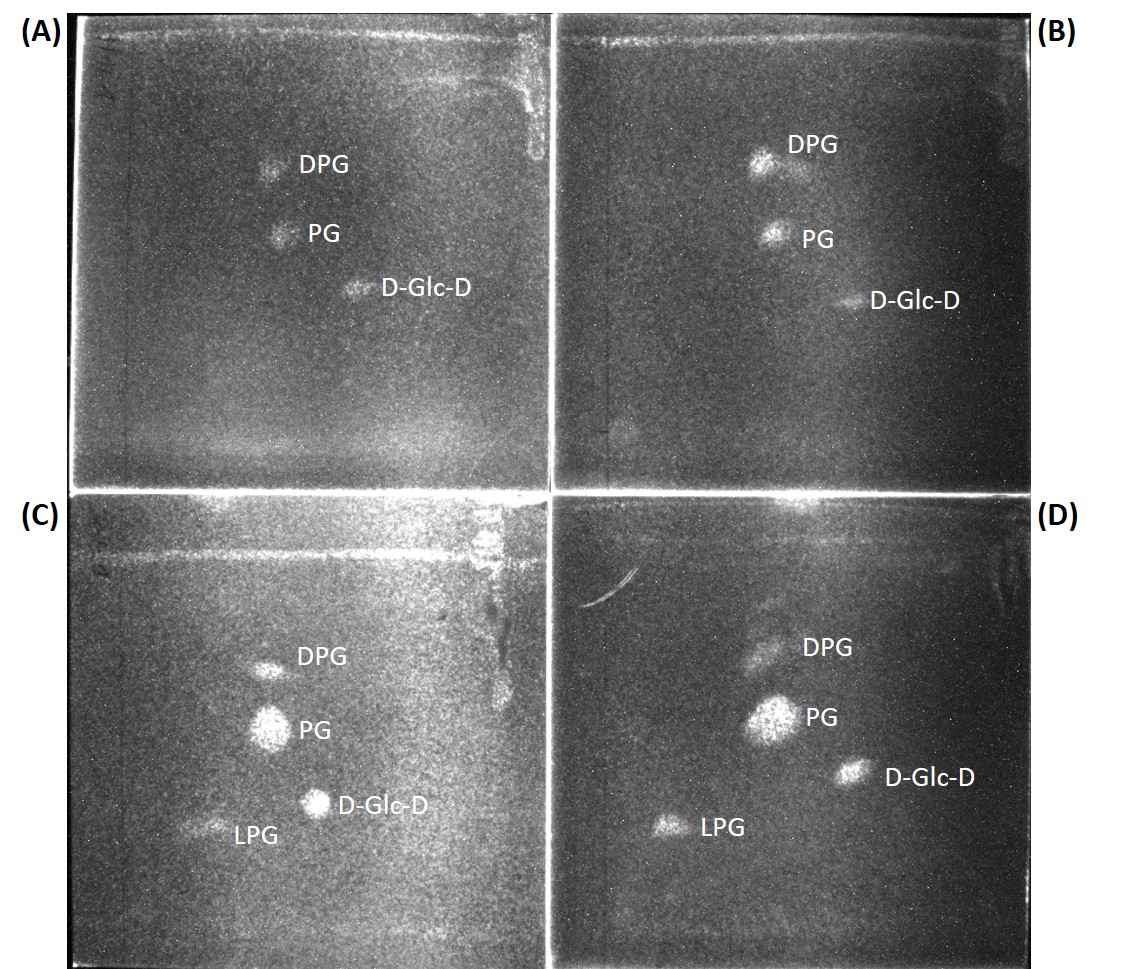


Figure 2 Two dimensional thin-layer-chromatography of polar lipids from *S. capitis* strains H17 (A), D2^T^ (B), K1 (C) and D3 (D). DPG, diphosphatidylglycerol; PG, phosphatidylglycerol; D-Glc-D, diglucosyldiacylglycerol; LPG, lysylphosphatidylglycerol.

# Predicted resistances based on genome

Table 6 Predicted resistances for antibiotics and heavy metals in *S. capitis* strains D2^T^, D3, K1 and H17 by AMRFinderPlus based on genomes. Gene names correspond to: fosfomycin resistance bacillithiol transferase FosB (*fosB*)*,* class A broad-spectrum beta-lactamase TEM (*blaTEM*), penicillinase repressor BlaI (*blaI*), beta-lactam sensor/signal transducer BlaR1 (*blaR1*), penicillin-hydrolyzing class A beta-lactamase BlaZ (*blaZ*), tetracycline efflux MFS transporter Tet(K) (*tet(K)*), arsenite efflux transporter membrane subunit ArsB/ArsC/ArsD (*arsB/arsC/arsD*), cadmium resistance transporter CadD (*cadD*).

| **D2^T^** | **D3** | **K1** | **H17** |
| --- | --- | --- | --- |
| *fosB* | *arsB* | *arsB* | *arsB* |
| *arsB* | *arsD* | *arsC* | *blaZ* |
| *arsC* | *cadD* | *arsB* | *blaR1* |
| *arsD* | *tet(K)* | *blaI* | *blaI* |
| *blaTEM* |  | *blaR1* |  |
|  |  | *blaZ* |  |
