# Supplementary figures and images for: Phenotypic and genomic assessment of the potential threat of human spaceflight-relevant Staphylococcus capitis isolates under stress conditions
Source: Front Microbiol. 2022 Nov 3;13:1007143. doi: 10.3389/fmicb.2022.1007143 (PMC9669719; doi:10.3389/fmicb.2022.1007143)

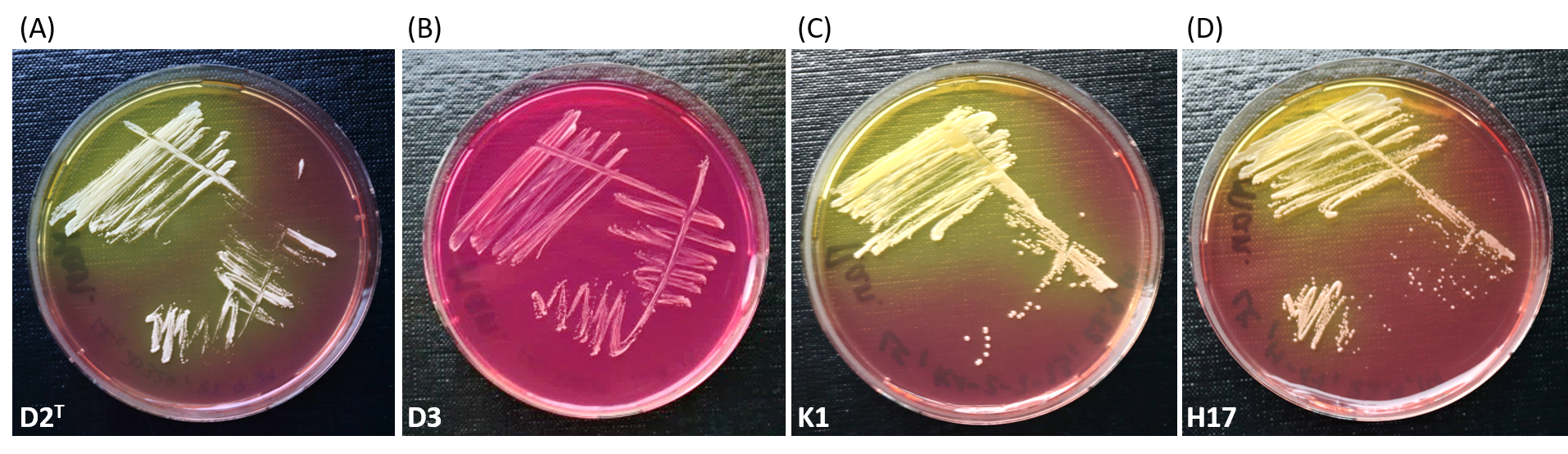

Supplement: Supplementary file 2 [file Image_1.JPEG]

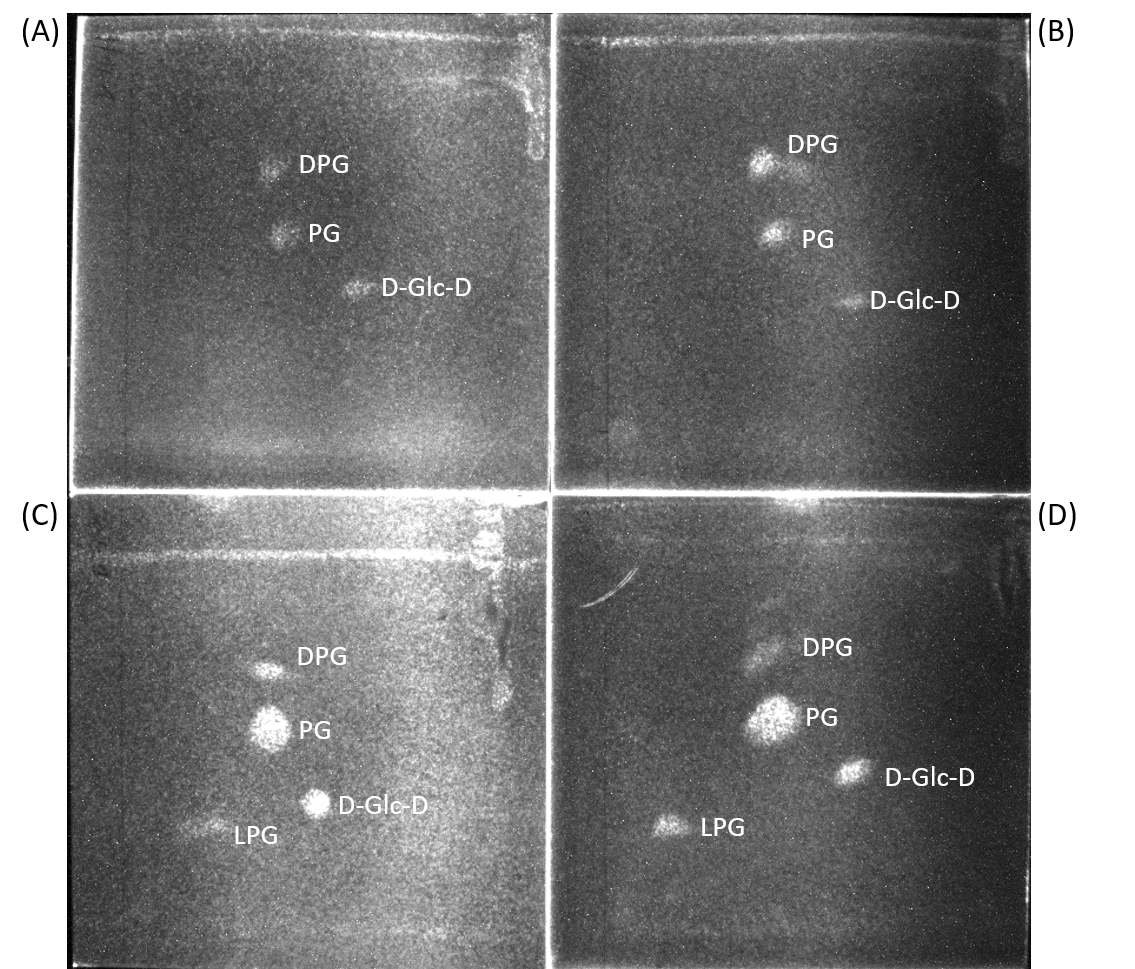

Supplement: Supplementary file 3 [file Image_2.JPEG]
